# Temporal associations supporting repetitions in free recall

**DOI:** 10.3758/s13423-025-02673-x

**Published:** 2025-04-24

**Authors:** Lynn J. Lohnas

**Affiliations:** Department of Psychology, Syracuse University, 765 Irving Avenue Suite 352, Syracuse, NY 13210 USA

**Keywords:** Episodic memory, Recall, Repetition effects, Spacing and lag effects

## Abstract

The present studies use a novel approach to characterize how memory representations are updated with repetition. These studies use the free recall paradigm, which boasts greater memory advantages for spaced repetitions (Melton. *Journal of Verbal Learning and Memory*, *9*, 596–606. 1970; Madigan. *Journal of Verbal Learning and Verbal Behavior*, *8*, 828–835. 1969). However, a single recall of a twice-presented item precludes inferring whether the item’s first or second presentation support its recall. The present studies leverage that, in free recall, transitions reflect stronger associations and are more likely between items studied nearby in time (Healey et al., *Psychonomic Bulletin & Review*, *26*(3), 699–720. 2019). The critical analysis asks which transitions are more likely to a repeated item: temporal neighbors from its first presentation or from its second presentation? Transitions should be equally likely from neighbors of each presentation if the repeated item’s presentations are stored independently. Transitions from second-presentation neighbors should be more likely if retrieval of item information from the first presentation strengthens the item representation during the second presentation, or if independent traces benefit from being studied more recently. Alternatively, retrieved context theory assumes that each studied item is associated with a slowly drifting temporal context, and repetition evokes study-phase retrieval of the context state from the first presentation (Howard & Kahana. *Journal of Mathematical Psychology*, *46*, 269–299. 2002a; Siegel & Kahana. *Journal of Experimental Psychology: Learning, Memory, and Cognition*, *40*(3), 755-764. 2014). This context retrieval should strengthen the repeated item’s associations to items with similar temporal contexts from its first presentation. As a result, retrieved context theory predicts more transitions to a repeated item from a first-presentation neighbor. Two studies provide support for the prediction of retrieved context theory, with implications for other theories.

**Supplementary Information:**

The online version contains supplementary material available at 10.3758/s13423-025-02673-x.

One does not need to research memory to intuit that information presented twice is generally remembered better than information presented once. A comprehensive explanation of this ubiquitous finding, termed the repetition effect, remains elusive. Resolving this debate has implications for how memories are represented and updated, as well as practical implications such as how to optimize repetition for improved memory. Cognitive accounts of the repetition effect center around the related spacing effect, the finding that memory for a repeated item increases with more intervening items between its presentations (Madigan, 1969; Melton, 1970; Donovan & Radosevich, 1999; Cepeda et al., 2006; Delaney et al., 2010).

Given the decades of debate and the shortcomings across multiple proposed accounts, more recently a consensus has emerged that two complementary accounts contribute to the repetition and spacing effects (Benjamin & Tullis, 2010; Delaney et al., 2010; Siegel & Kahana, 2014; Raaijmakers, 2003; Pavlik & Anderson, 2005; Malmberg & Shiffrin, 2005), although combining these accounts into an integrated theory has a long history (e.g., Glenberg, 1979). One critical component to this integrated theory is context — information surrounding but not comprising the memory itself. Particularly relevant for spaced items is temporal context, a type of context which changes slowly over time. Context variability theory, the first of two accounts incorporated into the integrated theory, posits that as the spacing increases between a repeated item’s presentations, its two associated temporal context states share less overlap. With a more diverse set of associated contexts, this in turn increases the probability that some context elements associated with the repeated item will be reactivated during the memory test to promote its recall (Bower, 1972; Estes, 1955; Melton, 1970).

The second component to the integrated theory makes stronger assumptions about the influence of repeating an item for its representation in memory. In particular, the study-phase retrieval account assumes that repetition serves to retrieve an item’s earlier presentation(s) (Greene, 1989; Hintzman, 2010; Thios & D’Agostino, 1976; Cuddy & Jacoby, 1982; Schmidt & Bjork, 1992). Taking these two accounts together into the integrated theory, termed retrieved context theory, the assumption is that the second presentation of an item retrieves the item’s context from its first presentation. Thus, the context associated with an item’s repetition is a recursive combination of the slowly drifting context and the item’s earlier context(s).

More recently, Siegel and Kahana (2014) examined predictions of retrieved context theory as implemented in a computational model of episodic memory, the context maintenance and retrieval model (CMR; Polyn et al., 2009). CMR assumes that each studied item is associated with a slowly changing temporal context, and context serves as the recall cue. When an item is experienced a second time, whether as a studied repetition or as a recall, this retrieves the item’s context from earlier presentations to update the temporal context state. In addition to predicting the repetition and spacing effect, CMR makes predictions relatively unique to the retrieved context account which have been found in experimental data. In CMR, each item serves as the input to context, and thus temporal context is a recency-weighted sum of past context states. As a result, if the temporal context from a repeated item’s first presentation is retrieved during its second presentation, this forges stronger connections between the items studied nearby the repeated item in both presentations. Thus, CMR accurately predicts more transitions between temporally distant items which neighbor a repeated item when compared to the same temporal transitions in lists of once-presented items (Siegel & Kahana, 2014). Taken together the retrieved context account provides a promising avenue for reconciling past results.

CMR has only simulated findings of the repetition effect and spacing effect in free recall. The spacing effect is present in many episodic memory tasks including item recognition and cued recall, but in free recall the effect is more robust and extends to larger spacings (e.g., Glenberg, 1976; Greene, 1989). However, there are some drawbacks to examining recall probability of repeated items in free recall, and more broadly this reflects a critical decision point in most study designs with repeated items. Studies which require participants to remember information based on each presentation of a repeated item, such as frequency judgments, may encourage participants to store separate representations of each presentation of the repeated item. By contrast, studies which require only a single report of the repeated item, such as free recall, may encourage participants to store a single memory representation. However, it is unclear whether a single recall of a repeated item reflects memory for all instances of the repeated item or only a subset. Even in the study of Siegel and Kahana (2014), the associations between items neighboring a repeated item hint at, but do not fully reveal, how context influences the representation of the repeated item.

The current studies present a new approach to assess how each of two presentations of a repeated item influence memory, without asking participants about either presentation of that item. Instead, using a free recall paradigm, associations with a repeated item are inferred based on the transition made to that item. Briefly, each critical list contains a single spaced, repeated item. If, as predicted by retrieved context theory, a participant retrieves temporal context from the first presentation when studying the second presentation, then this should strengthen the associations between the repeated item and the temporal context from its first presentation. This distant temporal context is most similar to the temporal contexts of items studied nearby in time to the first presentation. As a result, a temporal neighbor from the item’s first presentation, henceforth termed a first-presentation neighbor, should boast stronger associations than a second-presentation neighbor. Thus, transitions to the repeated item should be more likely from a first-presentation neighbor than a second-presentation neighbor. For instance, if a recalled item was studied at serial positions 6 and 12, then we can compare the proportion of recalls in which the transition to the repeated item comes from a first-presentation neighbor (serial positions 5 or 7) versus from a second-presentation neighbor (serial positions 11 or 13).

Critically, retrieved context theory, but not either of its component accounts in isolation, predict the advantage for recall transitions from first-presentation neighbors (Table 1). For instance, suppose context variability were present without study-phase retrieval. In this case, the context cue at the end of the recall period should more strongly cue recently studied items including the second repetition. Thus, according to this account second-presentation neighbors should be more likely to transition to the repeated item. Alternatively, if study-phase retrieval were present in the absence of context retrieval, then an item’s repetition should strengthen encoding of the item itself but not its associations to the temporal context from its first presentation. According to this item-based study-phase retrieval account, the recency of the second presentation should promote its recall.

**Table 1:** Predictions of Repetitions and Transitions by Account

Account	Retrieval at Repetition	More Transitions to Repetition from
Retrieved Context	Context	$1^{st}$ Presentation Neighbors
Context Variability	None	$2^{nd}$ Presentation Neighbors
Study-phase Retrieval	Item	$2^{nd}$ Presentation Neighbors
Deficient Processing	Item	$1^{st}$ or $2^{nd}$ Presentation Neighbors
Independent Traces	None	$2^{nd}$ Presentation Neighbors or Both Equally

Each account includes the type of information retrieved during a repetition, if any, as well as its predictions for whether transitions to a repeated item are from its $1^{st}$ or $2^{nd}$ presentation neighbors. For the Deficient Processing account, $1^{st}$ or $2^{nd}$ means that transitions may be more likely from one of the sets of presentation neighbors, depending on the assumptions of this account (as opposed to Both Equally). See text for details

Two other prominent accounts of repetition and spacing effects are also worth noting. According to the deficient processing account, participants devote less encoding to, or are more deficient in their processing of, items which are recognized as repetitions (Hintzman, 1974; Greene, 1989; Toppino et al., 2009). This account explains the spacing effect because an item repeated at a greater spacing is less likely to be recognized from its first presentation, and less likely to suffer from deficient processing. By comparison, their memory will be better on subsequent tests. In the present studies, a spaced repetition may still suffer from some deficient processing, and this would lead to weaker encoding of the second presentation. However, the present analyses are perhaps least diagnostic in assessing the deficient processing account, if one wishes to argue that deficient processing influences the repeated item’s associations to its temporal neighbors. On the one hand, the poor encoding of the repeated item could reduce its associations to its second-presentation neighbors. On the other hand, in the absence of encoding the repetition, second-presentation neighbors may benefit from additional encoding to help counteract the reduced associations.

As a final account, each presentation of the repetition might be encoded independently Hintzman (1984). All else being equal, both sets of presentation neighbors should serve as equally strong associative cues to the repeated item, and transitions would be equally likely from either set of presentation neighbors. Alternatively, the more recent presentation of the repetition may be represented more strongly in memory due its recency of occurrence, which in turn would make transitions from second-presentation neighbors more likely. Regardless, the predictions of this account stand in contrast to the prediction of retrieved context theory that transitions from first-presentation neighbors are more likely.

Note that the item supporting the repeated item, or the item transitioned *to* the repeated item, serves as the most diagnostic transition for this prediction of retrieved context theory. The item transitioned *from* the repeated item could reflect strengthened associations as well, but this transition suffers from other confounding factors. For instance, suppose that the transition is from the repeated item to a second-presentation neighbor. The most interesting case is when this transition reflects the item pair’s strengthened associations. However, instead this transition could reflect more weakly associated items simply because the strongest association already supported the transition to the repeated item. As another possibility, the transition from the repeated item may not be due to strengthened associations between the repeated item and the presentation neighbor, but rather the transition may reflect recall from a compound cue which incorporates the item recalled before the repeated item (e.g., Lohnas & Kahana, 2014, see Supplementary Materials).

Two studies examine the critical analysis of transitions to a repeated item from its neighbors at study. Experiment 1 includes a distractor task between study and the recall test, a common feature of repetition studies to reduce the recall advantage for items studied at the end of the list (Cepeda et al., 2006; Delaney et al., 2010). However, with such a distractor task participants tend to initiate recall at the start of the list (Howard & Kahana, 1999; Healey et al., 2014), and so this design may encourage recall from early list items including first-presentation neighbors of the repeated item. To this end, Experiment 2 uses nearly identical procedures to Experiment 1 but without an end-of-list distractor, as with this set-up participants tend to initiate recall with items studied most recently (Howard & Kahana, 1999; Healey & Kahana, 2014). To foreshadow the results, despite differences in recall initiation, both studies revealed that transitions were more likely from first-presentation neighbors to the repeated item, consistent with the retrieved context account.

## Method

### Experiment 1

#### Participants

The final set of included participants were 184 young adults (age 18–30) who were self-proclaimed native English speakers. An additional 23 participants were excluded from this final set. Of those, five participants were excluded for reporting on the post-study survey that they wrote down words during list presentation (preventing the recall test from fully relying on memory because they could view the words), and one participant was excluded for reporting on the post-study survey that they were not fully engaged with list presentation and the encoding task. Fourteen participants were excluded for typing responses all at once (without pressing enter), so their individual responses could not be parsed. Further, two participants were excluded because they did not follow task instructions and thus did not have enough correct responses to be included (one only typed only numbers and the other typed one correct recall with otherwise mostly numbers or symbols). Finally, one participant was excluded from analysis for not recalling any repeated items. This participant could not contribute to analyses of transitions to repeated items, and for consistency was excluded from all analyses.

#### Sampling procedures

Participants were recruited through Prolific. Recruitment, consent, experiment design and compensation procedures followed Prolific guidelines, had approval of Syracuse University’s Internal Review Board, and were in accordance with the 1964 Helsinki Declaration.

#### Data diagnosis

A planned power analysis conducted on the initial 50 participants indicated that 184 participants would be needed for power value of .80, at the $\alpha =.05$ level.

#### Materials

The materials in this study were the word lists which participants studied and recalled. Each of 10 lists was had 20 token words, taken from an initial larger pool of 576 words with defined semantic relatedness values using the Word Association Space (WAS) model described by Steyvers et al. (2004), as well as values of concreteness, valence and word frequency (Aka et al., 2021). All participants viewed the same 10 lists, to ensure similar recall across lists as well as to control for other variables of interest which may influence recall. In particular, because the critical analyses consider temporal transitions within each list, semantic similarity within lists was minimized to avoid competition from this type of association. Within a given list, semantic similarity WAS values between any pair of items was always less than 0.4 (cf. Long & Kahana, 2017). To further minimize recall differences across lists, each list had equivalent distributions of concreteness, valence and word frequency, such that for each word property, a one-way ANOVA with list as a factor yields $p>.2$.

Two of the lists, serving as control lists, had 20 unique items. The remaining eight lists had 19 unique items and one word presented twice. The repeated item was presented in one of four possible positions, each used twice for the eight lists: two first positions (6 or 7) X 2 number of intervening items (5 or 6). List order, and the order of words within each list, were randomized across participants. The variable placement of the repeated item in each list, and the random order of the lists with or without repeated items, aimed to minimize strategies to process or anticipate the repeated item.

To equate responses in the syllable-counting task (see next section), lists with an even number of words contained an equal number of 1-syllable words and 2-syllable words. Lists with an odd number of words were also approximately evenly divided: four lists had one more 1-syllable words than 2-syllable words, and four lists had one more 2-syllable words than 1-syllable words.

#### Procedure

Participants completed a single experiment session remotely on a web browser from their personal computer. The study procedure was coded and executed with jsPsych (de Leeuw, 2015). Participants began the study with instructions and a brief practice phase of an abbreviated list, distractor and recall period. Participants were given feedback if they didn’t recall any words during the practice period but were not given any feedback during the experiment itself. After the brief practice phase, they then began the main experiment of studying and recalling the 10 word lists. Each study list began with the presentation of a fixation cross for 3 s, followed by a blank interstimulus interval (ITI) of 0.5 s. Each word appeared on the screen for 3 s. To help maintain focus and minimize rehearsal, each word was presented with a task cue below it to indicate whether the word had 1 or 2 syllables. Participants could indicate their responses with the corresponding number on their keyboard. After the presentation of each word, a 0.5 s ITI either prompted the participant to respond faster during the if they did not respond within the 3 s word presentation, or was a blank screen if they did respond.

After the last ITI, participants performed a math distractor task, typing responses to arithmetic questions ($$A+B+C=?$$ where *A*, *B*, *C* were positive, single-digit integers). The distractor task was self-paced; once a participant entered a solution and the return key, a new problem appeared. After 16 s of the distractor task, a row of asterisks appeared for 0.5 s, and then a blank textbox appeared. Participants had 60 s to type any of the items from the most recent list in any order. After typing each word, the participant pressed enter which cleared the textbox. At the end of each recall period, participants could take a self-paced break, pressing a key to start the next list. After the final list, participants completed a survey indicating whether they wrote down words (which would go against the nature of the memory test) as well as addressing their effort and other distractions while completing the task remotely. Participants were then debriefed. The study took approximately 30 min to complete, and participants were compensated $6.

Participants’ typed responses were spellchecked manually without knowledge of the list types or conditions for each word.

### Experiment 2

Methods for Experiment 2 varied minimally from Experiment 1 with notable differences described below. In brief, Experiment 2 replaces the distractor period of Experiment 1 with more list items. Having a longer list-length in immediate free recall achieves two goals. First, this avoids having the second presentation of a repeated item and its neighbors benefit from the recency advantage for end-of list items, or recency effect. Second, because recall probability is generally higher in immediate than delayed free recall, the presentation of additional list items maintains approximately equal levels of recall between the two experiments. This in turn helps to ensure that differences in recall and transition probabilities between experiments reflect the change in distractor task alone.

#### Participants

The final set of participants were 156 young adults, none of whom completed Experiment 1. An additional fourteen participants were excluded from this final set: four were excluded for reporting on the post-study survey that they wrote down words during list presentation and nine were excluded for not pressing enter between recalled words. One participant was excluded from analysis for not recalling any repeated items. Because this participant could not contribute to analyses of transitions to repeated items, for consistency they were excluded from all analyses.

#### Data diagnosis

**Fig. 1:**
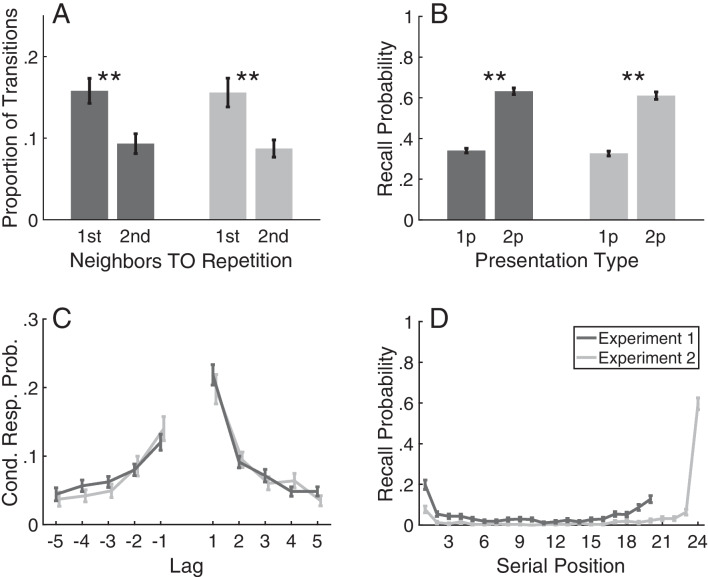
Recall Transitions and Recall Initiation. A: Recall transitions were more likely from a first-presentation neighbor than from a second-presentation neighbor. B: Recall probability for once-presented items (1p) and twice-presented items (2p) in lists with repeated items. C: Conditional response probability as a function of lag in lists without repeated items. For Experiment 2, this excludes the first three output positions (cf. Kahana, 1996). D: Probability of first recall in lists without repeated items. Errorbars show standard error of the mean. $$**=p<.01$$

A planned power analysis conducted on the initial 50 participants indicated that 156 participants would be needed for power value of.80, at the $\alpha =.05$ level. The somewhat smaller sample size for this experiment, in contrast to Experiment 1, might be a product of the more consistent recall initiation; whereas the majority of recall sequences in Experiment 2 begin with the final list item, the probability of first recall function is more uniform across serial positions for Experiment 1 (Fig. 1D). The consistency in recall patterns for Experiment 2 may in turn reduce how many participants need to contribute to get a significant effect.

#### Materials

The materials in this study were 10 lists of 24 token words, drawn from the same pool as in Experiment 1 (Aka et al., 2021). All participants viewed the same 10 lists, two of which were composed of all once-presented items and eight of which had a single word presented twice, repeated in the same serial positions as in Experiment 1.

#### Procedure

The procedure was identical to Experiment 1, except that there were no math distractor tasks. Thus, after the final ITI, a row of asterisks appeared for 0.5 s, and then a blank textbox appeared to initiate the recall period. As one other minor change based on the number of participants who were excluded from Experiment 1 for typing all of their responses at once, during the practice phase in Experiment 2 if a participant’s recalls contained a spacebar, they were also given feedback to not type the spacebar between words, but rather to press the enter key to submit one word at a time.

### Analyses

Planned power analyses and reported effect sizes used a variant of Cohen’s *d* based on the pooled standard deviation (Fritz et al., 2012).

## Results

For both experiments, in the lists with repeated items recall of twice-presented items was significantly greater than recall of once-presented items (Fig. 1B), Experiment 1: $M=0.632~vs.~0.340, SEM=0.0130, t(183)=22.4, p<.001, CI=[0.267,0.318], d=1.5$; Experiment 2: $M= 0.611~vs.~0.326, SEM = 0.0137, t(155)=20.7, p<.001, CI=[0.257,0.311], d=1.5$. Critically, in both experiments participants were significantly more likely to transition to a repeated item from a first-presentation neighbor than from a second-presentation neighbor (Fig. 1A), Experiment 1: $M=0.158~vs.~0.0932, SEM=0.0213, t(183)=3.05, p=0.0026, CI=[0.0229,0.107], d=0.34$; Experiment 2: $M=0.156~vs.~0.0872, SEM=0.0223, t(155)=3.09, p=0.0024, CI=[0.0247,0.113], d=0.38$. These results are consistent with the retrieved context account assumption that the temporal context of a repeated item’s first presentation is retrieved during its second presentation, which in turn strengthens the repeated item’s associations to its first-presentation neighbors.

Each participant performed recall of two lists without repeated items to ensure that recall behavior replicates prior findings in two important ways. First, they ensure that participants made temporal transitions consistent with prior studies. Figure 1C conveys temporal transitions as a function of lag, or difference in serial positions. Participants exhibited greater transition probabilities between items with smaller absolute values of lag, termed the temporal contiguity effect (Healey et al., 2019; Kahana, 1996). Importantly, the use of temporal information in recall transitions should benefit recall of repeated items. A second important replication in the lists without repeated items concerns probability of first recall as a function of serial position (Fig. 1D; Healey et al., 2014; Howard & Kahana, 1999). Notably, in Experiment 2 participants were more likely to recall recently presented items, but in Experiment 1 the distractor reduces the accessibility of end-of-list items. Despite the differences in recall initiation, however, both studies favored associations to the repeated item from first-presentation neighbors.

Differences in recall across serial and output positions, as well as the pattern of temporal transitions, may nonetheless bias transitions towards first-presentation neighbors of the repeated item. To rule out this possibility, I examined the proportion of temporal transitions in lists without repeated items yet at serial positions matched to repeated items (Supplementary Materials). Using data sets with similar methods and results to the present studies but with more lists per participant, differences in temporal transitions were not significant, $p's>.05$. Taken together, this suggests that the repeated items are critical for producing the increased temporal transitions from first-presentation neighbors.

## Discussion

The present studies query memory representations of repeated items by leveraging the rich history of inferring associations from recall transitions (e.g., Bhatarah et al., 2008; Kahana, 1996; Lohnas & Kahana, 2014; Long et al., 2015; Sadeh et al., 2015). In two free recall studies with different recall initiation patterns, transitions were more likely to a repeated item from—thus suggestive of stronger associations with—its first-presentation neighbors than its second-presentation neighbors. These results are most consistent with theories assuming that the second presentation of the repeated item evokes study-phase retrieval to strengthen its associations with its first-presentation neighbors.

This explanation follows naturally from retrieved context theory, which assumes that each studied item is associated with a slowly changing temporal context and repeating an item evokes retrieval of earlier temporal contexts (Siegel & Kahana, 2014; Raaijmakers, 2003; Glenberg, 1979; Delaney et al., 2010; Malmberg & Shiffrin, 2005; Pavlik & Anderson, 2005). Thus, the second presentation of a repeated item retrieves the context from its first presentation, which in turn strengthens the repeated item’s associations to items with similar temporal contexts to its first presentation. In the Supplementary Materials, Simulations of retrieved context models—across varied parameter values and secondary assumptions—demonstrate the importance of the assumption of context retrieval for greater cue strengths of first- than second- presentation neighbors. In general, first-presentation neighbors exhibit stronger cue strengths when a repeated item retrieves more strongly the temporal context immediately after its first presentation, and by incorporating the updated temporal context into the item-context associations of the repeated item. However, for some model variants with high rates of both context drift and context retrieval, the item after the second occurrence of the repeated item has strong associations with both temporal contexts of the repeated item and thus boasts greater cue strength. Nonetheless, in general across model variants and parameter values, first-presentation neighbors have greater cue strengths.

Rather these results suggest that the reinstatement of context is critical to any study-phase retrieval account. Similarly, a deficient processing account would need to ensure that the deficiency in processing of the second presentation of the repeated item does not lead to extra encoding in temporal associations of second-presentation neighbors. Both accounts could assume that temporal context or item associations strengthen not only the repeated item but also its associations to first-presentation neighbors.

Empirically, the current results build on previous free recall studies which suggest that retrieval of context information from first presentations strengthens associations between items related to each repetition (Howard et al., 2007; Sederberg et al., 2011; Siegel & Kahana, 2014; Rait et al., 2023). Yet the present studies provide a more direct form of evidence that this context retrieval supports recall of repeated items.

Although the results are consistent with retrieved context theory, they still only provide a piece of a larger puzzle, and other factors may contribute to the role of temporal context in the repetition effect. For instance, there may be limitations in memory ability or variability in the amount of temporal context retrieved across items (e.g., Sadeh et al., 2015). In addition, despite the attempts to minimize semantic similarity, semantic associations may nonetheless compete with temporal associations to support recall of repeated items (Healey & Uitvlugt, 2019; Howard & Kahana, 2002b; Bousfield et al., 1958; Romney et al., 1993; Pollio et al., 1969). These results will also need to be reconciled with findings suggesting that context retrieval plays less of a role in repetition effects (e.g., Adams & Delaney, 2023). Future work will need to consider experiment manipulations or analyses based on unique predictions of these accounts.

It would also be informative to leverage methods beyond free recall behavior, such as cognitive neuroscience studies which query representations of repeated items with brain activity (e.g., Heusser et al., 213; Lohnas et al., 2018; Manelis et al., 2011; Turk-Browne et al., 2012; Yassa & Stark, 2011). In addition, requiring participants to perform overt rehearsal during list presentation would elucidate for each list and participant whether the second presentation of an item evokes rehearsal of its first-presentation neighbors (Rundus, 1971; Ward & Tan, 2023). This would inform how endogenous retrieval influences subsequent encoding and recall transitions. Another important avenue in task instructions include those which more strongly encourage distinguishable representations of the repeated item. Free recall arguably places fewer constraints on how the repeated item is represented, but the requirement to recall each item once could encourage an integrated representation across presentations. If this were the case, the present results suggest that temporal context from the first presentation weighs more heavily in this representation.

Despite the open questions and remaining future work, the present results help to provide a piece of the puzzle of the representation of repeated items in the absence of strong task instructions. They underscore how study-phase retrieval of associative information can support memory for spaced repetitions.

## Supplementary Information

Below is the link to the electronic supplementary material.

Supplementary file 1 (pdf 1132 KB)

## Data Availability

The data and materials for all experiments are available in the Open Science Framework repository at https://osf.io/4n7ch/.

## Code availability

Study-specific code is available in the Open Science Framework repository at https://osf.io/4n7ch/. These analyses relied on code available from https://github.com/vucml/EMBAM.

## Declarations

### Open Practices Statement

The data and materials for all experiments are available in the Open Science Framework repository at https://osf.io/4n7ch/ and none of the experiments was preregistered.
