# Supplementary material for: Temporal associations supporting repetitions in free recall
Source: Psychon Bull Rev. 2025 Apr 24;32(5):2211–9. doi: 10.3758/s13423-025-02673-x (PMC12426128; doi:10.3758/s13423-025-02673-x)
Supplement: Supplementary file 1 — (pdf 1132 KB) [file 13423_2025_2673_MOESM1_ESM.pdf]

Supplementary Materials for
Temporal associations supporting repetitions in free recall

Lynn J. Lohnas

Overview

This document aims to supplement analyses and simulations to link CMR predictions to the results reported in the main text, through two sets of results. The first set of results is more theoretical, providing a more rigorous mathematical account of the predictions of retrieved context models for a repeated item to be cued by its first-presentation and second-presentation neighbors. This section first derives model equations then evaluates the equations across a range of parameter sets and model assumptions. For instance, the degree to which each item encodes or retrieves context are controlled by several model parameters; if these model parameters are set so that context plays a small role, then it is possible for the model to predict that the second-presentation neighbors will cue the repetition more strongly. However, overall this section shows that across the majority of parameter values and model variants, pairwise comparisons favor the first-presentation neighbors as being stronger cues of the first-presentation neighbors over the second-presentation neighbors.

The second set of results provides empirical control analyses for the serial position and transition effects reported in the main text. This section first reports more detailed analyses of serial position and transitions to ensure that confounds in these variables cannot explain the current results. Next, additional analyses of other studies with more expansive sets of control lists provide a control of the potential influence of serial position, output position and conditional response probability across successive transitions. In brief, at matched serial positions transitions in these lists, transitions are not significantly different from first-presentation or second-presentation neighbors, suggesting that the item repetition in the present studies promotes transitions from earlier serial positions.

Retrieved context model predictions of cuing from presentation neighbors of a repetition

We begin by summarizing how items are encoded into the model and recalled from the model. Next, we review several model mechanisms which contribute to a neighbor to cue a repetition, all founded in the intuition that the repeated item's contexts serve as its strongest cue. We consider these mechanisms across several model variants, and two components always play a role: (1) the similarity between the neighbor's retrieved context and the repeated item's context; (2) the similarity between the current state of context and the repeated item's contexts. In addition, in some model variants pre-experimental episodic

context-to-item associations also contribute to the similarity of the repeated item and its presentation neighbors.

Retrieved context model assumptions of repetitions during study

Most broadly, retrieved context models assume that each studied or recalled item updates a context state, and the current context state is the recall cue, and that temporal context contributes to this context representation. To isolate the role of these context representations, we do not assume that other forms of context contribute to the retrieval cue. We also begin by assuming that other assumptions follow the context and retrieval model, which has been tested most extensively directly to repetition effects (Lohnas Siegel & Kahana, 2014; Polyn et al., 2009).

We consider temporal context to be represented as a vector, such that context state from an item i is denoted $\mathbf{t}_i$ and the ith element of $\mathbf{t}$ is denoted $\mathbf{t}(i)$.

Equally important to temporal context representations are how these representations interact with studied and recalled items. Each item is represented as a vector of features $\mathbf{f}$. The $\mathbf{t}$ and $\mathbf{f}$ vectors interact through associative matrices $\mathbf{M}^{FT}, \mathbf{M}^{TF}$ where the first letter of the superscript corresponds to the input vector and the second letter corresponds to the output. Each matrix is a weighted sum of a pre-experimental component and an experimental component, with the latter component updated during presentation and recall of the experiment. Model parameters γ_{FT}, γ_{TF} scale the relative amount of experimental and pre-experimental contributions to the respective matrices $\mathbf{M}^{FT}, \mathbf{M}^{TF}$. These parameters take on values in the range [0,1], and larger values reflect a greater contribution of experimental context. For the remainder of this document, for simplicity we will refer to γ_{FT} as γ. Importantly:

$$\mathbf{M}^{FT} = (1-\gamma)\mathbf{M}^{FT}_{pre} + \gamma\mathbf{M}^{FT}_{exp}. \tag{S1}$$

When an item j is studied, this activates its associated feature vector $\mathbf{f}_j$, and this creates an input to update context:

$$\mathbf{t}^{\mathbf{IN}}_j = \frac{\mathbf{M}^{FT}\mathbf{f}_j}{||\mathbf{M}^{FT}\mathbf{f}_i||}. \tag{S2}$$

This input updates context according to the equation:

$$\mathbf{t}_j = \rho_j \mathbf{t}_{j-1} + \beta \mathbf{t}^{\mathbf{IN}}_j, \tag{S3}$$

where ρ_j changes with each input to context weighted so that $||\mathbf{t}_j|| = 1$:

$$\rho_j = \sqrt{1 + \beta^2[(\mathbf{t}_{j-1} \cdot \mathbf{t}^{\mathbf{IN}}_j)^2 - 1]} - \beta(\mathbf{t}_{j-1} \cdot \mathbf{t}^{\mathbf{IN}}_j). \tag{S4}$$

The β parameter controls the degree to which context updates with each presented item. Larger values of β cause context to reflect more recently studied items, at the cost of downweighing prior context states.

First presentation

When an item $\mathbf{f}_i$ is studied for the first time, this simplifies the above equations for two reasons. First, CMR, like most retrieved context models to date, represents items using a localist representation, such that

$$\mathbf{f}_i(j) = \begin{cases} 1 & \text{if } j = i \\ 0 & \text{otherwise.} \end{cases} \tag{S5}$$

Further, $\mathbf{M}^{FT}_{pre}$ is initialized to an identity matrix, and $\mathbf{M}^{FT}_{exp}$ is initialized to a matrix of zeroes. As a result, when applying Equation S2, the ith set of elements in $\mathbf{M}^{FT}$ are 0 except element i, and so $\mathbf{t}^{\mathbf{IN}}_i = \frac{(1-\gamma)\mathbf{f}_i}{1-\gamma} = \mathbf{f}_i$.

This also simplifies Equation S4, because of Equation S5 yet $\mathbf{t}_{i-1}(i) = 0$. So, the term $\mathbf{t}_{i-1} \cdot \mathbf{f}_i = \mathbf{t}_{i-1} \cdot \mathbf{t}^{\mathbf{IN}}_i = 0$. We can thus rewrite Equation S3 as

$$\mathbf{t}_i = \rho_i \mathbf{t}_{i-1} + \beta \mathbf{f}_i = \sqrt{1-\beta^2}\mathbf{t}_{i-1} + \beta \mathbf{f}_i. \tag{S6}$$

The model then stores the association between the studied item and the context state using an outer Hebbian product. In CMR this updating follows:

$$\Delta \mathbf{M}^{TF}_{exp} = \mathbf{f}_i \mathbf{t}_i^\top \tag{S7}$$

$$\Delta \mathbf{M}^{FT}_{exp} = \mathbf{t}_i \mathbf{f}_i^\top \tag{S8}$$

We first consider the associations as in the above equation but note how this changes with varying assumptions in the subsequent sections.

Having defined the representations and associations from studying an item for the first time, we next define some other critical properties between studied items. Assuming the items in positions i, j have each been presented once, then:

$$\mathbf{f}_i \cdot \mathbf{t}_j = \begin{cases} \beta \rho_i^{j-i} & \text{if } j \geq i \\ 0 & \text{otherwise,} \end{cases} \tag{S9}$$

$$\mathbf{t}_i \cdot \mathbf{t}_j = \rho_i^{|j-i|}. \tag{S10}$$

Note that $\rho_i = \rho_j$ (see EquationS6), but we keep this subscript for specificity in the second presentation, which we turn to next.

Second presentation

When item i is presented for the second time, this activates the same set of item features from its first presentation, $\mathbf{f}_i$. However, its associated context states now differ at this new list position, denoted with the subscript r. As with the first presentation, this feature vector creates an input to context (Equation S2), and the ith element has a pre-experimental component weighted by $1-\gamma$. However, for the second presentation this input to context contains an experimental component from the first presentation of $\mathbf{f}_i$, based on its association to $\mathbf{t}_i$ (Equations S1, S8):

$$\mathbf{t}_r^{\mathbf{IN}} = k[(1-\gamma)\mathbf{f}_i + \gamma\mathbf{t}_i], \tag{S11}$$

where

$$k = (2(1-\beta)\gamma^2 - 2(1-\beta)\gamma + 1)^{-1/2} \tag{S12}$$

(To derive k, which essentially the inverse of $||\mathbf{t}_r^{\mathbf{IN}}||$, we can use Equation S5 to infer that this vector is essentially $\gamma\mathbf{t}_i$ with the addition in i^{th} element of $1-\gamma$. If $\mathbf{t}_i(i) = \beta$ and $||\mathbf{t} = 1||$, then it must be the case that $\sum_{j=1}^{i-1}\mathbf{t}_i(j) = \sqrt{1-\beta^2}$. Thus, $\mathbf{t}_r^{\mathbf{IN}}(i)$ is a sum of $\gamma\beta$ from $\mathbf{t}_i$ and $1-\gamma$ from $\mathbf{f}_i$. In total then,

$$||\mathbf{t}_r^{\mathbf{IN}}|| = (\gamma\sqrt{1-\beta^2} + \beta\gamma^2 + 1 - \gamma)^2 \tag{S13}$$

We can then derive k from expanding and rearranging terms.)

Now using this input to update context, using Equation S3:

$$\begin{aligned}\mathbf{t}_r &= \rho_r\mathbf{t}_{r-1} + \beta\mathbf{t}_r^{\mathbf{IN}} \\ &= \rho_r\mathbf{t}_{r-1} + k\beta(1-\gamma)\mathbf{f}_i + k\beta\gamma\mathbf{t}_i,\end{aligned} \tag{S14}$$

where

$$\rho_r = \sqrt{1+\beta^2[(\mathbf{t}_{r-1}\cdot\mathbf{t}_r^{\mathbf{IN}})^2 - 1]} - \beta(\mathbf{t}_{r-1}\cdot\mathbf{t}_r^{\mathbf{IN}}). \tag{S15}$$

In Equation S14, the context vector $\mathbf{t}_r$ is a weighted sum of three vectors, each reflecting a critical assumption of retrieved context models:

1. $\mathbf{t}_{r-1}$ demonstrates the core assumption of these models that context is a recency-weighted sum of prior context states. This also ensures that the second presentation of the repeated item is associated with items studied nearby in time, despite retrieval of context from the first presentation. It is always the case that $\rho_r < \rho_i$ due to the greater similarity between the input to context and the prior context state than when item i is preceded by a completely novel set of items.

2. $\mathbf{f}_i$ reflects retrieval of item features from the repeated item's first presentation. This serves to associate the features of item i with the second presentation as well.

3. $\mathbf{t}_i$ demonstrates context retrieval from the repeated item's first presentation. This last term distinguishes retrieved context models from item-based models and can be further intuited from its weights. As noted above, γ refers to γ_{FT}, a free model parameter ranging between 0 and 1, and smaller values produce smaller contributions of experimental context. In the extreme case that $\gamma = 0$, then the $\mathbf{t}_i$ is multiplied by zero, effectively eliminating context retrieval. Similarly, if context does not drift with each studied item, such that $\beta = 0$, then context retrieval does not occur either. Taken together, the assumption of context retrieval for a repeated item holds at nearly all parameter values.

Comparison of contexts of first and second presentations during recall

As described above, the current context state is used to cue recall of an item. The critical question here is whether first-presentation neighbors or second-presentation neighbors cue the repeated item more strongly. To determine this, we will first walk through the logic of re-presenting a neighbor of a repeated item to the model. Then, we will discuss how this item's input serves to cue context, which is ultimately quantified with a single number of cue strength. We can compare this number across all four of the presentation neighbors to get a sense of how often each of the presentation neighbors has the greatest cue strength value, and how this varies with model parameters.

The same equations governing item presentation during study also apply to recall of an item, inasmuch as a recalled item is presented endogenously. Thus, Equation S11 defines the input to context for a presentation neighbor just after the model recalls it. For simplicity we consider the case where the presentation neighbor is the first recalled item.

We distinguish between an item's initial input to context and its generated context from recall with $'$, e.g., $\mathbf{t}_{i-1}^{\mathbf{IN}}$ and $\mathbf{t}_{i-1}^{\prime\mathbf{IN}}$ respectively. This input produces an updated state of context, which we assume occurs x items after item r:

$$\mathbf{t}'_w = \rho_{r+x}\mathbf{t}_{r+x-1} + \beta\mathbf{t}_w^{\prime\mathbf{IN}}, \tag{S16}$$

where

$$\mathbf{t}_w^{\prime\mathbf{IN}} = k[(1-\gamma)\mathbf{f}_w + \gamma\mathbf{t}_w]. \tag{S17}$$

.

This input to context then updates context according to Equation S3 to yield a set of activation values:

$$\mathbf{a}_w = \mathbf{M}^{TF}\mathbf{t}'_w, \tag{S18}$$

where $\mathbf{a}$ is a vector of activation values, one element for each studied item. For each possible presentation neighbor $w \in \{i-1, i+1, r-1, r+1\}$, we are interested in how

strongly it cues the repeated context based on the context it generates. Thus, for the current discussion, we are interested in the value of $\mathbf{a}_w(i)$—effectively the cue strength for the feature of the repeated item $\mathbf{f}_i$—from each of the four presentation neighbors w. In particular, we are interested in whether $\mathbf{a}_w(i)$ from first-presentation neighbors exceed those of second-presentation neighbors.

The value of $\mathbf{a}_w(i)$ depends on the dot product similarity of the repeated item's contexts and the current context based on the neighbor, i.e., $\mathbf{t}'_w \cdot \mathbf{t}_i + \mathbf{t}'_w \cdot \mathbf{t}_r$. We can divide this similarity into three components:

1. The $\mathbf{f}_w$ component of $\mathbf{t}'^{\mathbf{IN}}_w$ (see Equations S16, S17,Table S1).[1]
2. The $\mathbf{t}_w$ component of $\mathbf{t}'^{\mathbf{IN}}_w$ (see Equations S16, S17,Table S1).
3. The weighted previous state of context $\rho_{r+x}\mathbf{t}_{r+x-1}$ (i.e., the first term on the right hand side of Equation S16; see also Equation S4). The scalar ρ_{r+x} which weighs this context state depends on $\mathbf{t}_{r+x-1} \cdot \mathbf{t}'^{\mathbf{IN}}_w$, and this term is calculated in Table S2. However, the degree to which the current state of context cues the repeated item's context is the same for all presentation neighbors:

$$\mathbf{t}_{r+x-1} \cdot (\mathbf{t}_i + \mathbf{t}_r) = \rho_i^{x-1}(1 + \rho_r \rho_i^{r-i-1} + \beta^2(1-\gamma)k + \beta\gamma k). \tag{S19}$$

Taken together the components depend on β and γ. Although the equations in Tables S1, S2 include other variables as well, we can redefine most variables in terms of β and γ. For k and ρ_i, see respectively Equations S12 and S6. For ρ_r, we can use Equation S15, replacing $\mathbf{t}^{\mathbf{IN}}_r$ based on Equation S11:

$$\begin{aligned}
\mathbf{t}_{r-1} \cdot \mathbf{t}^{\mathbf{IN}}_r &= \mathbf{t}_{r-1} \cdot (k(1-\gamma)\mathbf{f}_i + k\gamma\mathbf{t}_i) && \text{(S20)}\\
&= k(1-\gamma)\beta\rho_i^{r-i-1} + k\gamma\rho_i^{r-i-1}\\
&= k(1-\beta^2)^{0.5(r-i-1)}(\beta(1-\gamma)+\gamma), && \text{(S21)}
\end{aligned}$$

where the final line replaces $\rho_i = \sqrt{1-\beta^2}$ (Equation S4).

We will also need to set two variables which do not depend on β or γ. First, the value for the spacing between repetitions $r - i = 6$ (a possible value from the experiments). Next, we will set the value of x, the distance between the second presentation r and when the presentation neighbor is presented again to the model, which contributes to ρ_{r+x}. We first consider $x = 10$, which in Experiment 2 approximates when the presentation neighbor was the first recalled item of the list. We will also consider other values for $r - i, x$ after first considering these initial values.

[1] In all tables, the terms were first derived by hand, then verified by running model simulations with the standard above equations and comparing the model output to the equations.

Table S1

Similarity Between Context of Repeated Item and Input to Context from Presentation Neighbors

w	$\mathbf{f}_w \cdot \mathbf{t}_i$	$\mathbf{f}_w \cdot \mathbf{t}_r$	$\mathbf{t}_w \cdot \mathbf{t}_i$	$\mathbf{t}_w \cdot \mathbf{t}_r$
$i-1$	$\beta\rho_i$	$\beta\rho_r\rho_i^{r-i} + k\beta^2\gamma\rho_i$	ρ_i	$\rho_r\rho_i^{r-i} + k\beta\gamma\rho_i$
$i+1$	0	$\beta\rho_r\rho_i^{r-i-2}$	ρ_i	$\rho_r\rho_i^{r-i-2} + k\beta^2(1-\gamma)\rho_i + k\beta\gamma\rho_i$
$r-1$	0	$\beta\rho_r$	ρ_i^{r-i-1}	$\rho_r + k\beta^2(1-\gamma)\rho_i^{r-i-1} + k\beta\gamma\rho_i^{r-i-1}$
$r+1$	0	0	$\rho_r\rho_i^{r-i} + k\beta^2(1-\gamma)\rho_i + k\beta\gamma\rho_i$	ρ_i

Note. Each row corresponds to one of the four neighbors w of the item repeated at i and r, $w \in \{i-1, i+1, r-1, r+1\}$. Each column corresponds to different components contributing to calculating the similarity between the item's input to context and the repeated item's context, based on the first two items listed on the previous page and divided into two context states of the repeated item $(\mathbf{t}_i, \mathbf{t}_r)$.

Table S2

Similarity Between Feature of Presentation Neighbors to Prior Context

w	$\mathbf{t}_{r+x-1} \cdot \mathbf{t}_w'^{\mathbf{IN}}$
$i-1$	$\rho_i^{x-1}[(1-\gamma)k(\beta\rho_r\rho_i^{r-i} + \beta^2\gamma k\rho_i) + \gamma k(\rho_r\rho_i^{r-i} + \beta\gamma k\rho_i)]$
$i+1$	$\rho_i^{x-1}[(1-\gamma)k\beta\rho_r\rho_i^{r-i-2} + \gamma k(\beta^2(1-\gamma)k\rho_i + \rho_r\rho_i^{r-i-2} + \beta\gamma k\rho_i)]$
$r-1$	$\rho_i^{x-1}[(1-\gamma)k\beta\rho_r + \gamma k(\beta^2(1-\gamma)k\rho_i^{r-i-1} + \rho_r + \beta\gamma k\rho_i^{r-i-1})]$
$r+1$	$\rho_i^{x-2}[(1-\gamma)k\beta + \gamma k]$

Note. Each row corresponds to one of the four neighbors w of the item repeated at i and r, $w \in \{i-1, i+1, r-1, r+1\}$. For simplicity, instead of calculating ρ_{r+x} this table provides $\mathbf{t}_{r+x-1} \cdot \mathbf{t}_w'^{\mathbf{IN}} = \zeta_{r+x}$, such that $\rho_{r+x} = \sqrt{1 + \beta^2(\zeta_{r+x}^2 - 1)} - \beta\zeta_{r+x}$ (Equation S4).

We are finally ready to consider simulations evaluated across a variety of parameter values. We consider $\beta, \gamma \in [.1, .9]$ because both parameters can range from [0,1] but it is rare for model simulations to have values close to the extremes. Even for both of the boundaries of this more limited range, model parameters rarely if ever take on both extreme values at once (from viewing several papers of which report best-fit parameter values from an algorithm search, Lohnas et al., 2015; Pazdera & Kahana, 2023; Polyn et al., 2009; Sederberg et al., 2008). Figure S1 plots the difference in cue strengths, $\mathbf{a}_w$, for each pairwise comparison of presentation neighbors (i.e., values of w). Blue indicates that the first-presentation neighbor of interest more strongly cues the repeated item, and darker values indicate a stronger cue from one of the presentation neighbors.

As a common theme among these pairwise comparisons, when the context drift rate β is low then the difference is smaller for the stronger cue strengths of first-presentation over second-presentation neighbors, for two reasons. First, less of the repeated item's context from i ($\mathbf{t}_i$) is retrieved during its second presentation at r (i.e., the furthest right term in Equation S16). This in turn reduces the similarity between the context at r ($\mathbf{t}_r$) with the context of its first-presentation neighbors ($\mathbf{t}_{i-1}, \mathbf{t}_{i+1}$). With this core assumption of retrieved context models set to more of a minimum, this in turn minimizes the advantage of first-presentation neighbors. A second reason that low β values reduce cue strengths of first-presentation neighbors concerns its impact on the context at $r-1$. If $\beta = 0$, then temporal context plays a minimal role in distinguishing the representations of the repeated item. Similarly, when context drifts very slowly then the contexts between the two presentations of the repeated items are relatively similar, more closely approximating item-based representations. This also benefits the items surrounding the second presentation of the repeated item, as now the representations between repetitions are highly similar, and more minimally reflect the assumption of a drifting context rate.

Only the comparison of $i+1$ to $r-1$ yields values for which the second-presentation neighbor cues the item more strongly. This occurs for less than 30% of possible values considered, specifically for higher values of β and lower values of γ. With lower values of γ, less context from the first presentation of the repeated item is retrieved during its second presentation. Yet when β is higher, context drifts further between repetitions, and will be further from the context near i at the time of recall without the benefit of stronger reinstatement. As a result, $r-1$ will cue the repeated item more strongly due to more of its context being present in the current context state. Higher values of β and lower values of γ also reflect weaker cuing from the first-presentation neighbor between $i-1$ versus $r-1$, h, although this is not enough for $r-1$ to serve as a weaker cue. Most likely, the advantage for $i-1$ more than $i+1$ reflects the forward asymmetry advantage of $i-1, r-1$ being presented before the item repeated at i, r.

By contrast, comparing the cue strength of first-presentation neighbors to $r+1$, when γ is high this means that item $r+1$ will have more of the context of item i, which makes this item a better cue for the repeated item. Nonetheless, this is not enough to counter the strong cue strength of item $i-1$.

It is also worth noting these (and subsequent) simulations assume that β has a single value. However, in most simulations of retrieved context models, β has separate values for encoding and retrieval (β_{enc} and β_{rec}, respectively). Future work may also consider that the second presentation of an item updates context at a different rate than its first presentation

Figure S1

Difference in Context Cue Strength of Presentation Neighbors

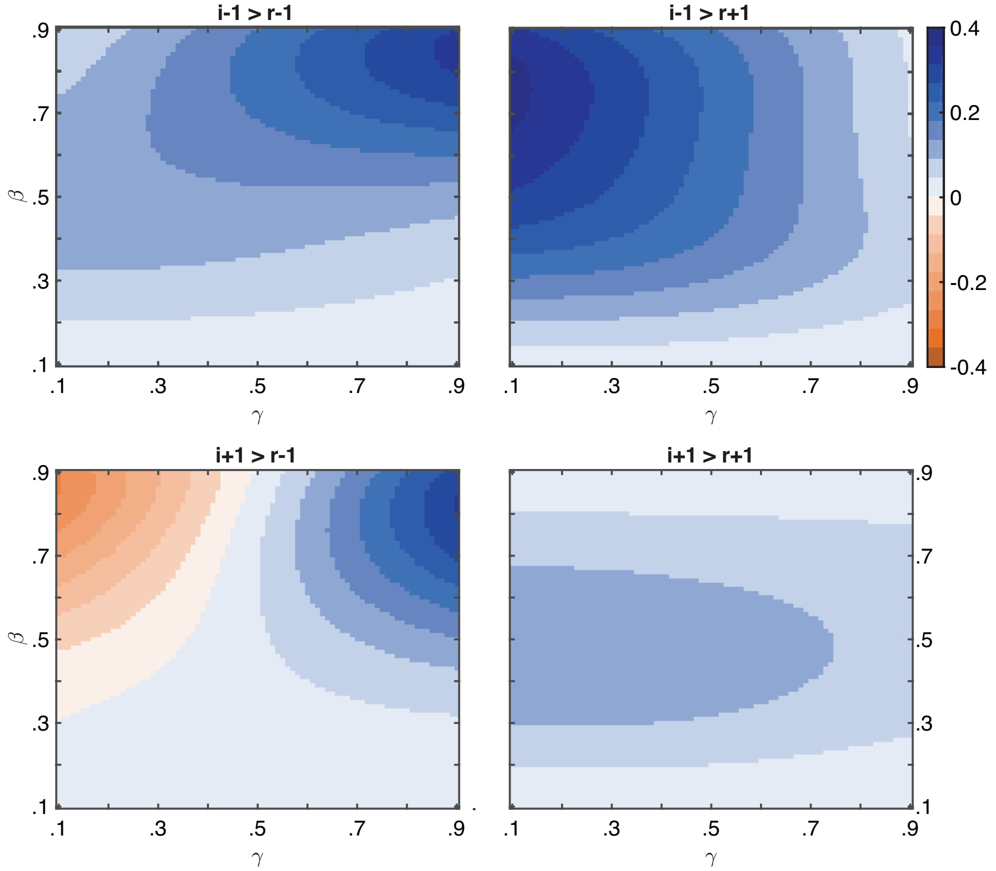

Note. The cue strength of the repeated item $(\mathbf{a}_w)$ from each presentation neighbor (i.e., $w \in \{i-1, i+1, r-1, r+1\}$) was calculated based on presenting the repeated item six items apart, then presenting a presentation neighbor ten items later. Each plot shows the difference in cue strength of a pair of first versus second-presentation neighbors across a range of values for β, γ. The color bar indicates the numerical difference of the cue strength for the first minus second-presentation neighbor: Blue indicates a greater cue strength from the first than second-presentation neighbor, and orange indicates a greater cue strength from the second- than first-presentation neighbor.

Figure S2

Difference in Context Cue Strength by Lag

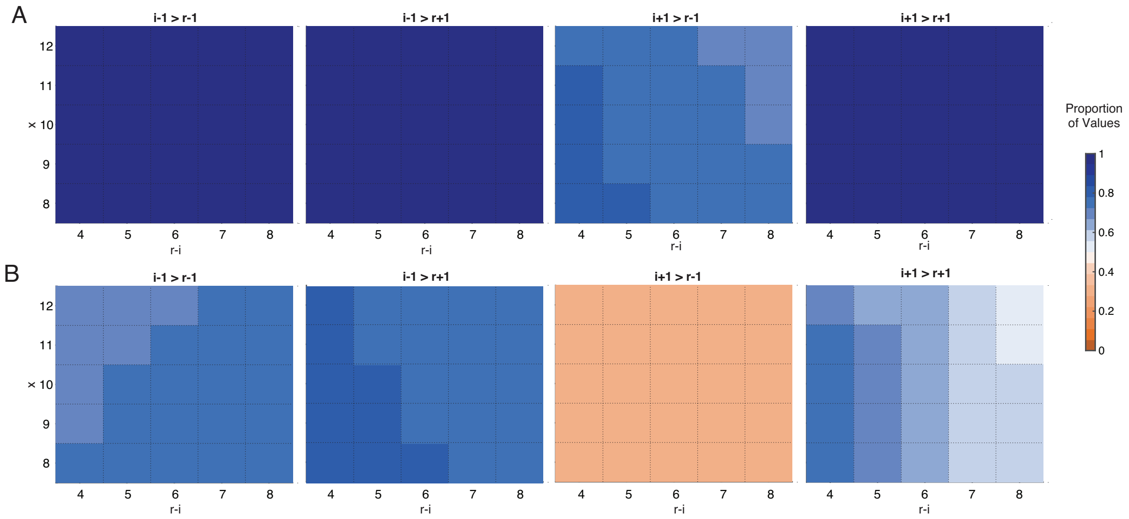

Note. Each panel plots the proportion of parameter sets with cue strengths satisfying the inequality at the top of the graph, varied across $\beta, \gamma \in [0.1, 0.9]$. Blue indicates a majority of values are greater for the first-presentation neighbors. (A) Simulations with $\Delta \mathbf{M}^{TF}_{exp} = (\Delta \mathbf{M}^{FT}_{exp})^{\top} = \mathbf{f}_i \mathbf{t}_i^{\top}$. (B) Simulations with $\Delta \mathbf{M}^{TF}_{exp} = (\Delta \mathbf{M}^{FT}_{exp})^{\top} = \mathbf{f}_i \mathbf{t}_{i-1}^{\top}$.

(e.g., Gershman et al., 2012). Varying β across these conditions complicates both the preceding equations and the interpretative power to a more prohibiting degree. Importantly, each presentation neighbor updates context to the same degree when it serves as the retrieval cue, and so the plots generally still give a sense of the variability in associations based on values of β.

These plots also assumed a fixed value for the lag between the first and second presentations of the repeated item, $r - i$, and the lag between the second presentation of the repeated item, x. Figure S2A plots the proportion of comparisons which yield an advantage for first-presentation over second-presentation neighbors, across all (6561) values of $\beta, \gamma \in [0.1, 0.9]$ in 0.1 increments from Figure S1, for values of $r - i \in \{4, 5, 6, 7, 8\}$ and $x \in \{8, 9, 10, 11, 12\}$. The choice of lag does not lead to drastic changes, and for half of the comparisons changing these variables has a minimal impact on the proportion of cue strengths which are greater for the first-presentation neighbor. When they do vary, in general larger lag values lead to a greater proportion of parameter sets with greater cue strengths for the second-presentation neighbors, even though the majority are still greater for first-presentation neighbors. Larger values of x reduce contributions of $\mathbf{t}_{i-1}, \mathbf{t}_{i+1}$ to $\mathbf{t}_x$, which in turn influence how strongly these first-presentation neighbors can cue $\mathbf{t}_i, \mathbf{t}_r$. Similarly, larger values of $r - i$ decrease the amount of overlap between $\mathbf{t}_{i-1}, \mathbf{t}_{i+1}$ with $\mathbf{t}_r$, and thus later reduce the cue strength of these first-presentation neighbors.

Repetition in other model variants

Associating items and context

In some retrieved context models, each item $\mathbf{f}_j$ is not associated with the current state of context $\mathbf{t}_j$ but rather with the prior context state, $\mathbf{t}_j$ (e.g., Lohnas et al., 2015; Sederberg et al., 2008). The presentation of item i updates the association matrices as:

$\Delta\mathbf{M}^{TF}_{exp} = (\Delta\mathbf{M}^{FT}_{exp})^\top = \mathbf{f}_i\mathbf{t}^\top_{i-1}$

Thus, the second presentation of the repetition retrieves $\mathbf{t}_{i-1}$ not $\mathbf{t}_i$:

$$\mathbf{t}^{\mathbf{IN}}_r = k_1[(1-\gamma)\mathbf{f}_i + \gamma\mathbf{t}_{i-1}], \tag{S22}$$

$$\mathbf{t}_r = \rho_r\mathbf{t}_{r-1} + \beta\mathbf{t}^{\mathbf{IN}}_r \tag{S23}$$

$$= \rho_r\mathbf{t}_{r-1} + k_1\beta(1-\gamma)\mathbf{f}_i + k_1\beta\gamma\mathbf{t}_{i-1}, \tag{S24}$$

where

$$k_1 = (2\gamma^2 - 2\gamma + 1)^{-1/2}. \tag{S25}$$

To derive k_1, we can use the fact that $||\mathbf{f}_i|| = ||\mathbf{t}_i|| = 1$. Equally important, the nonzero elements of $\mathbf{f}_i$ and $\mathbf{t}_i$ do not overlap, because $\mathbf{t}_i(j) \neq 0$ only when $j = i$, yet $t_i(j) \neq 0 \forall j < i$. Therefore, $||\mathbf{t}^{\mathbf{IN}}_r|| = \sqrt{(1-\gamma)^2 + \gamma^2}$, and rearranging terms gives Equation S25.

Conceptually, the most important difference for this model variant is how the repeated item's context is associated with other items. Because each item feature is associated to the previous context state, when item i is repeated $\mathbf{f}_i$ becomes associated with $\mathbf{t}_{r-1}$, and the context state at the time of the repeated item, $\mathbf{t}_r$, is associated with $\mathbf{f}_{r+1}$. Thus, the repeated item's second context, $\mathbf{t}_{r-1}$, does not incorporate the retrieved context at $\mathbf{t}_r$. Rather, the increased associations between the contexts of first-presentation neighbors and the repeated item is based on the contexts of the first-presentation neighbors incorporated into current state of context.

These differences to the associations between item features and contexts also have implications for two other sets of values. First, recalculating from Equation S19:

$$\mathbf{t}_{r+x-1} \cdot (\mathbf{t}_{i-1} + \mathbf{t}_{r-1}) = \rho_r\rho_i^{r-i+x-1} + \beta\gamma k_1\rho_i^{x-1} + \rho_r\rho_i^{x-1} + \beta^2 k_1(1-\gamma)\rho_i^{r-i+x-2} + \beta\gamma k_1\rho_i^{r-i+x-1}$$
$$= \rho_i^{x-1}[(\beta k_1\gamma + \rho_r)(1 + \rho_i^{r-i}) + \beta^2 k_1(1-\gamma)\rho_i^{r-i-1}]$$

Second, recalculating from Equation S20:

$$\mathbf{t}_{r-1} \cdot \mathbf{t}^{\mathbf{IN}}_r = \mathbf{t}_{r-1} \cdot (k_1(1-\gamma)\mathbf{f}_i + k_1\gamma\mathbf{t}_{i-1}) \tag{S26}$$

$$= k_1(1-\gamma)\beta\rho_i^{r-i-1} + k\gamma\rho_i^{r-i}$$

$$= k_1(1-\beta^2)^{0.5(r-i-1)}(\beta(1-\gamma) + \gamma\sqrt{1-\beta^2}). \tag{S27}$$

We will once again need to determine the extent to which each of the first and second-presentation neighbors cue the repeated item's associated context, now for $\mathbf{t}_{r-1} + \mathbf{t}_{i-1}$

Figure S3

Difference in Context Cue Strength of Presentation Neighbors

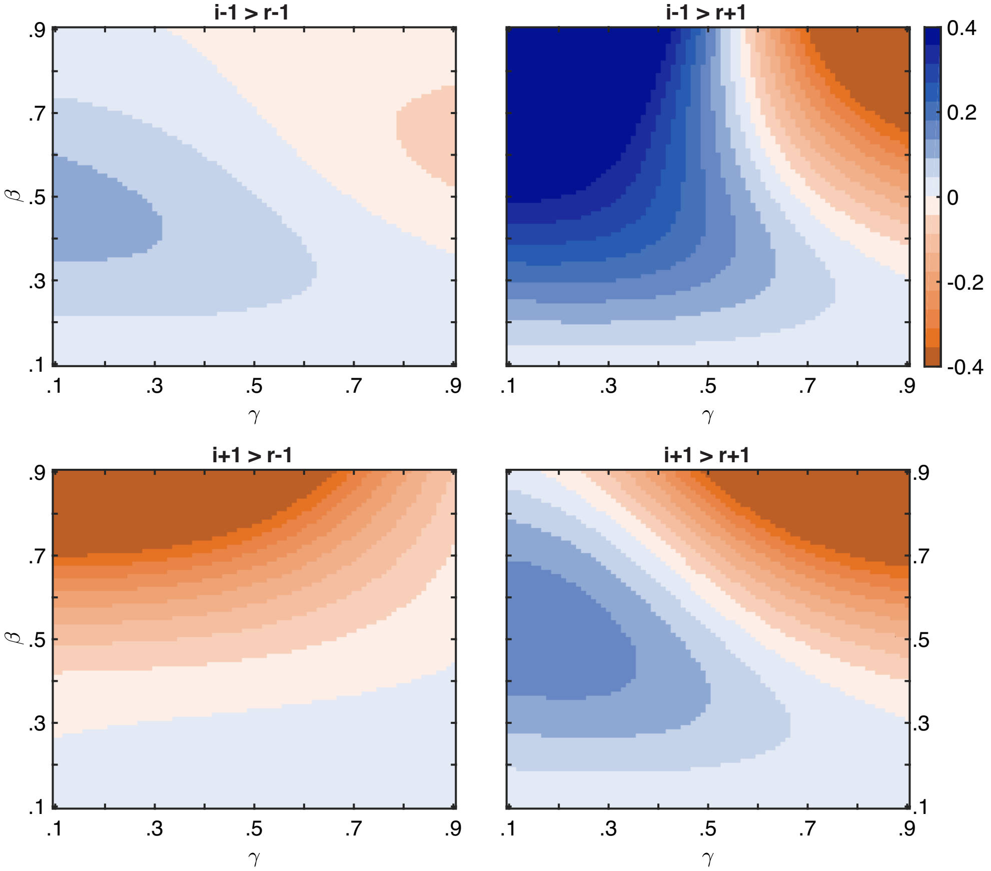

Note. The cue strength of the repeated item from each presentation neighbor was calculated based on presenting the repeated item six items apart, then presenting a presentation neighbor ten items later. Each plot shows the difference in cue strength of a pair of first versus second-presentation neighbors across a range of values for β, γ. The color bar indicates the numerical difference of the cue strength for the first- minus second-presentation neighbor: Blue indicates a greater cue strength from the first than second-presentation neighbor, and orange indicates a greater cue strength from the second- than first-presentation neighbor.

Table S3

Similarity Between Context of Repeated Item and Input to Context from Presentation Neighbors

w	$\mathbf{f}_w \cdot \mathbf{t}_{i-1}$	$\mathbf{f}_w \cdot \mathbf{t}_{r-1}$	$\mathbf{t}_w \cdot \mathbf{t}_{i-1}$	$\mathbf{t}_w \cdot \mathbf{t}_{r-1}$
$i-1$	β	$\beta\rho_i^{r-i}$	ρ_i	ρ_i^{r-i+1}
$i+1$	0	$\beta\rho_i^{r-i-2}$	ρ_i	ρ_i^{r-i-1}
$r-1$	0	β	ρ_i^{r-i-1}	ρ_i
$r+1$	0	0	$\rho_r\rho_i^{r-i} + k_1\beta\gamma$	$\rho_r + k_1\beta^2(1-\gamma)\rho_i^{r-i-1} + k_1\beta\gamma\rho_i^{r-i}$

Note. Each row corresponds to one of the four neighbors w of the item repeated at i and r, $w \in \{i-1, i+1, r-1, r+1\}$. Columns corresponds to the first two components contributing to calculating the similarity between the item's input to context and the repeated item's context, divided into two context states of the repeated item $(\mathbf{t}_{i-1}, \mathbf{t}_{r-1})$.

Table S4

Similarity Between Feature of Presentation Neighbors to Prior Context

w	$\mathbf{t}_{r+x-1} \cdot \mathbf{t}_w'^{\mathbf{IN}}$
$i-1$	$\rho_i^{x-1}[(1-\gamma)k_1\beta\rho_i^{r-i-1} + \gamma k_1(\rho_r\rho_i^{r-i+1} + \beta\gamma k_1\rho_i)]$
$i+1$	$\rho_i^{x-1}[(1-\gamma)k_1\beta\rho_r\rho_i^{r-i-2} + \gamma k_1(\rho_r\rho_i^{r-i-1} + \beta^2(1-\gamma)k_1 + \beta\gamma k_1\rho_i)]$
$r-1$	$\rho_i^{x-1}[(1-\gamma)k_1\beta\rho_r + \gamma k_1(\rho_r\rho_i + \beta^2(1-\gamma)k_1\rho_i^{r-i-2} + \beta\gamma k_1\rho_i^{r-i-1})]$
$r+1$	$\rho_i^{x-2}[(1-\gamma)k_1\beta + \gamma k_1\rho_i]$

Note. Each row corresponds to one of the four neighbors w of the item repeated at i and r, $w \in \{i-1, i+1, r-1, r+1\}$. For simplicity, instead of calculating ρ_{r+x} this table provides $\mathbf{t}_{r+x-1} \cdot \mathbf{t}_w'^{\mathbf{IN}} = \zeta_{r+x}$, where $\rho_{r+x} = \sqrt{1+\beta^2(\zeta_{r+x}^2 - 1)} - \beta\zeta_{r+x}$.

(Tables S3,S4). Similarly, for each of the four comparisons of interest ($i-1$ vs. $r-1$, $i+1$ vs. $r+1$, $i+1$ vs. $r-1$, $i+1$ vs. $r+1$), we will consider the weighted sum of the inputs with the similarity to the current context (see Equation S24; Figure S3). In general, when compared to the previous set of simulations (Figure S1) the proportion and magnitude of differences in the advantage of first-presentation neighbors is reduced. For all comparisons, this reflects that the repeated item r is not associated with the context from its first presentation i but rather with the prior state of context $r-1$. Thus, the benefit of reinstated context is not present in direct item-to-context and context-to-item associations, but rather it is present by incorporating $\mathbf{t}_{i-1}$ to the current context. At the time of recall, the context cue thus still reflects strengthened associations between the contexts associated with the repeated item and those of first presentation neighbors. As another contributing factor the weakened advantage of first-presentation neighbors for this model variant, because $\mathbf{f}_{r+1}$ is associated with $\mathbf{t}_r$ it is a more effective cue for the repeated item (as opposed to the previous model simulations in which $\mathbf{f}_{r+1}$ was associated to $\mathbf{t}_{r+1}$). Nonetheless, for three out of four comparisons the first-presentation neighbors serve as a stronger retrieval cue in the majority of cases (over $\sim 70\%$ of values with $i-1$ as the first-presentation neighbor, and over $\sim 60\%$ between $i+1$ and $r+1$).

Like the previous model variant, in the present variant the effects are weakest for the comparison between $i+1$ and $r-1$, with $\sim 70\%$ of parameter values yielding a stronger

cue strength for the second-presentation neighbor $r-1$. These results are again weakest when β is high and γ is low, following the intuition of the previous section. Nonetheless, for the parameter sets where the second-presentation neighbor is strongest in this comparison, there is an advantage of first-presentation neighbors in the other three comparisons. For these three simulations, cue strengths for second-presentation neighbors are greatest when context retrieval and context drift are both high. At these more extreme values, this serves a bit less like incorporating some of $\mathbf{t}_{i-1}$ into $\mathbf{t}_r$, and more like overwriting $\mathbf{t}_r$ with $\mathbf{t}_{i-1}$. Combined with context drifting further away from $\mathbf{t}_{i-2}$,$\mathbf{t}_i$ and the strengthened associations between $\mathbf{t}_{i-1}, \mathbf{t}_{r-1}$ present in the current context state, this leads to weaker cue strength for the first-presentation neighbors. This is probably less problematic for $i-1$ than $i+1$ because retrieval of $\mathbf{t}_{i-1}$ incorporates the context of $i-1$ but not $i+1$.

Also like the previous set of simulations, low values of β produce a smaller difference in cue strength for first- over second-presentation neighbors. The intuition is the same whether context is associated to the previous or current state of context. In brief, lower β values yield a reduced rate of context retrieval for a repeated item, thus reducing the shared associations between the repeated item and its first-presentation neighbors. Further, low β values increase the similarity of the contexts between a repeated item's occurrence, functioning more like an item-based account than a retrieved context account. This in turn increases the similarity between the second-presentation neighbors to both occurrences of the repeated item.

Whereas the above simulations show simulations for $r-i=6, x=10$, Figure S2B shows how these cue strength differences vary across other values of $r-i, x$. All values favor recall of the first-presentation neighbors across the four pairwise comparisons in Figures S3, except for the comparison of $i+1$ to $r-1$, yet the disadvantage remains relatively stable across lag values. The intuition for reduced cue strength of first-presentation neighbors at larger lags is the same as the previous model variant.

Pre-experimental context-to-item associations

In some model variants, there is a trade-off with the amount of pre-experimental versus experimental context contributing to the context-to-item associative matrix:

$$\mathbf{M}^{TF} = (1-\gamma_{TF})\mathbf{M}^{TF}_{pre} + \gamma_{TF}\mathbf{M}^{TF}_{exp}, \tag{S28}$$

whereas thus far we have been assuming that $\gamma_{TF} = 1$. However, if $0 < \gamma_{TF} < 1$, then applying Equation S18, now $\mathbf{M}^{TF}_{pre}$ contributes to the recall cue. Assuming that $\mathbf{M}^{TF}_{pre}$ is an identity matrix (e.g., Sederberg et al., 2008), when multiplying $\mathbf{M}^{TF}$ by the context state to cue item i, the similarity values calculated in the previous section (Tables S1,S2) are now weighed by γ_{TF}, and are summed with the similarity of each presentation neighbor to the nonzero element of $\mathbf{M}^{TF}$ at (i,i) (Table S5), weighed by $1-\gamma_{TF}$.

We first consider contributions of $\mathbf{M}^{TF}_{pre}$ for the original version of the model, where $\Delta\mathbf{M}^{TF}_{exp} = \mathbf{f}_i\mathbf{t}_i^\top$. Now we will need to consider three variables: γ_{TF}, β, and γ_{FT} (the latter of which, for short, in all equations has been γ). Rather than infer the similarities across three variables at once, I provide the plots of differences between presentation neighbors with $\beta \in \{.1, .3, .5, .7, .9\}$ (Figure S4). In brief, as γ_{TF} gets smaller, the current context state

Table S5

Similarity Between Feature of Repeated Item and Context States Assuming $\Delta M^{TF}_{exp} = \mathbf{f}_i \mathbf{t}_i^{\top}$

w	$\mathbf{f}_i \cdot \mathbf{t}_{r+x}$
$i-1$	$\rho_{r+x}(\rho_i^{x-2}\beta\rho_r\rho_i^{r-i} + \beta k(1-\gamma+\beta\gamma)\rho_i)$
$i+1$	$\beta^2\gamma k\rho_i + (\rho_{r+x}\rho_i^{x-1}[\beta k(1-\gamma+\beta\gamma) + \beta\rho_r\rho_i^{r-i-1})]$
$r-1$	$\beta^2\gamma k\rho_i^{r-i-1} + \rho_{r+x}\rho_i^{x-1}(\beta k(1-\gamma+\beta\gamma) + \beta\rho_r\rho_i^{r-i-1})$
$r+1$	$\beta\gamma k(\gamma\rho_i k\beta^2 + \beta\rho_i k(1-\gamma) + \beta\rho_i\rho_i^{r-i})+$ $\rho_{r+x}\rho_i^{x-1}(\beta k(1-\gamma+\beta\gamma) + \beta\rho_r\rho_i^{r-i-1})$

plays less of a role in which item will be recalled next, and thus reduces the contributions of associations from first-presentation neighbors. Thus, generally reduced values of γ_{TF} show less advantage for the first-presentation neighbor, aside from the difference between $i+1$ and $r-1$. In that case, when less of $\mathbf{t}_i$ is reinstated during $\mathbf{t}_r$ (due to lower γ) and context drifts more rapidly away from $i-1$ (due to higher β), then this more closely approximates an item-based study-phase retrieval account. Thus, having more item strength of the repeated item incorporated into $\mathbf{t}_r$ then benefits how much $r-1$ can cue $\mathbf{t}_r$.

Yet for much of the parameter space, temporal context contributes a sufficient amount to be consistent with the experimental findings. Indeed, several aspects of this figure mirror those of Figure S1, and at least 50% of the simulations favor first-presentation over second-presentation neighbors in all of these examples. In addition to the relationship between $i+1$ and $r-1$ as described in the previous paragraph, the cue strength of $r-1$ is reduced for high values of β and γ, because the repeated item's context drifts context further away from $r-1$ yet reinstates more of the original context. Also like both sets of previous simulations, lower values of β produce weaker cue strengths of first-presentation neighbors, even though these cue strengths may still favor the first-presentation neighbors. In general, the reduced difference, i.e. weaker cues of first-presentation neighbors, is even more pronounced when γ is low as well, as this further reduces the degree to which $\mathbf{t}_i$ is retrieved at $\mathbf{t}_r$.

Next, we also consider the model variant which combines the two previous model variants, as this model variant is used often as well (e.g., Lohnas et al., 2015; Pazdera & Kahana, 2023; Sederberg et al., 2008). Given that both model variants—associating the item to the previous state of context and setting γ_{TF}<1—reduce the proportion of parameter sets which predict an advantage for first-presentation neighbors, it is perhaps unsurprising that this combined model variant has even fewer parameter sets which predict this advantage. The patterns of differences are also in line with those of incorporating each mechanism separately: higher values of β cause context to drift further away from the context associated with the first presentation and higher values of γ more strongly associate such context to the neighbors of the second presentation.

Indeed, when β is very large (0.9), more than half of the cue strength values are greater for second-presentation neighbors, and mostly this is when γ, γ_{TF} are large. On the one hand, this subset of parameters might seem like the ideal conditions for the use of context retrieval and storing contextual information, because more context from $\mathbf{t}_{i-1}$ is incorporated into $\mathbf{t}_r$ when all three parameters have high values. On the other hand, such

Table S6

Similarity Between Feature of Repeated Item and Context States Assuming $\Delta M^{TF}_{exp} = \mathbf{f}_i \mathbf{t}^{\top}_{i-1}$

w	$\mathbf{f}_i \cdot \mathbf{t}_{r+x}$
$i-1$	$\rho_{r+x}\rho_i^{x-1}(\beta k_1(1-\gamma)+\beta\rho_r\rho_i^{x-i-1})$
$i+1$	$\gamma k_1\beta^2+\rho_{r+x}\rho_i^{x-1}(\beta k_1(1-\gamma)+\beta\rho_r\rho_i^{x-i-1})$
$r-1$	$\gamma k_1(\beta^2\rho_i^{x-i-2})+\rho_{r+x}\rho_i^{x-1}(\beta k_1(1-\gamma)+\beta\rho_r\rho_i^{x-i-1})$
$r+1$	$\beta\gamma k_1[\beta\rho_r\rho_i^{x-i-1}+\beta k_1(1-\gamma)]+\rho_{r+x}\rho_i^{x-1}(\beta k_1(1-\gamma)+\beta\rho_r\rho_i^{x-i-1})$

high values of $\beta, \gamma, \gamma_{TF}$ may effectively cause $\mathbf{t}_{i-1}$ to overwrite $\mathbf{t}_r$, and thus this benefits the second-presentation neighbors as well. In addition, smaller values of γ_{TF} reduce the contribution of the pre-experimental context, but the pre-experimental context of item $i-1$ more strongly cues $\mathbf{t}_{i-1}$.

When $\beta = 0.5$ or 0.7, more than half of cue strengths are greater for the second-presentation neighbor than for $i-1$. Larger context drift causes even the context associated with the first presentation of the repeated item ($\mathbf{t}_{i-1}$) to be further away from the context of $i-1$ ($\mathbf{t}_{i-2}$), and thus $\mathbf{t}_{i-2}$ will be more weakly reinstated when the repeated item is presented at r. However, for all other comparisons more than half of the cue strength values are greater for the first-presentation neighbor.

The common theme also still holds that there are generally low differences in cue strength with lower values of β. Moreover, like the immediately preceding set of simulations, low values of γ compound with the low values of β, overall reducing context retrieval for the repeated item. This is in line with the predictions of retrieved context models, that when its core assumptions are at more of a minimum—whether with more minimal context drift or minimal context retrieval—then this in turn minimizes its prediction of increased cue strengths for first-presentation neighbors.

Similar to when $\gamma_{TF} = 1$, these values are mostly consistent across lags yet can be weaker at larger lags. In Figure S6 each square corresponds to the proportion of values favoring first-presentation neighbors for $\beta, \gamma, \gamma_{TF} \in [0.1, 0.9]$ at 0.01 increments. Although the proportion of transitions does favor $r-1$ over $i-1$ when $\Delta\mathbf{M}^{TF}_{exp} = (\Delta\mathbf{M}^{FT}_{exp})^{\top} = \mathbf{f}_i\mathbf{t}^{\top}_{i-1}$, these strengths are not necessarily large (e.g., leftmost panels of Figure S5).

Summary

We considered the advantage of first- over second-presentation neighbors across four model variants (whether an item feature is associated with the current or previous context state X whether pre-experimental context-to-item associations contribute to the context cue). Three primary model parameters controlled the cue strengths for the presentation neighbors: the context drift rate β, the relative strength of item-to-context associations γ_{FT} (termed γ for short), and the relative strength of context-to-item associations γ_{TF}. Two experimental parameters also influenced the results: the lag $r-i$ between the item repeated at positions r and i, as well as the lag between the second repetition and the second occurrence of the presentation neighbor, x.

In the preceding six plots, blue colors indicate an advantage for first- over second-presentation neighbors, and the results generally favor the former. Across all sets of as-

Figure S4

Difference in Context Cue Strength of Presentation Neighbors

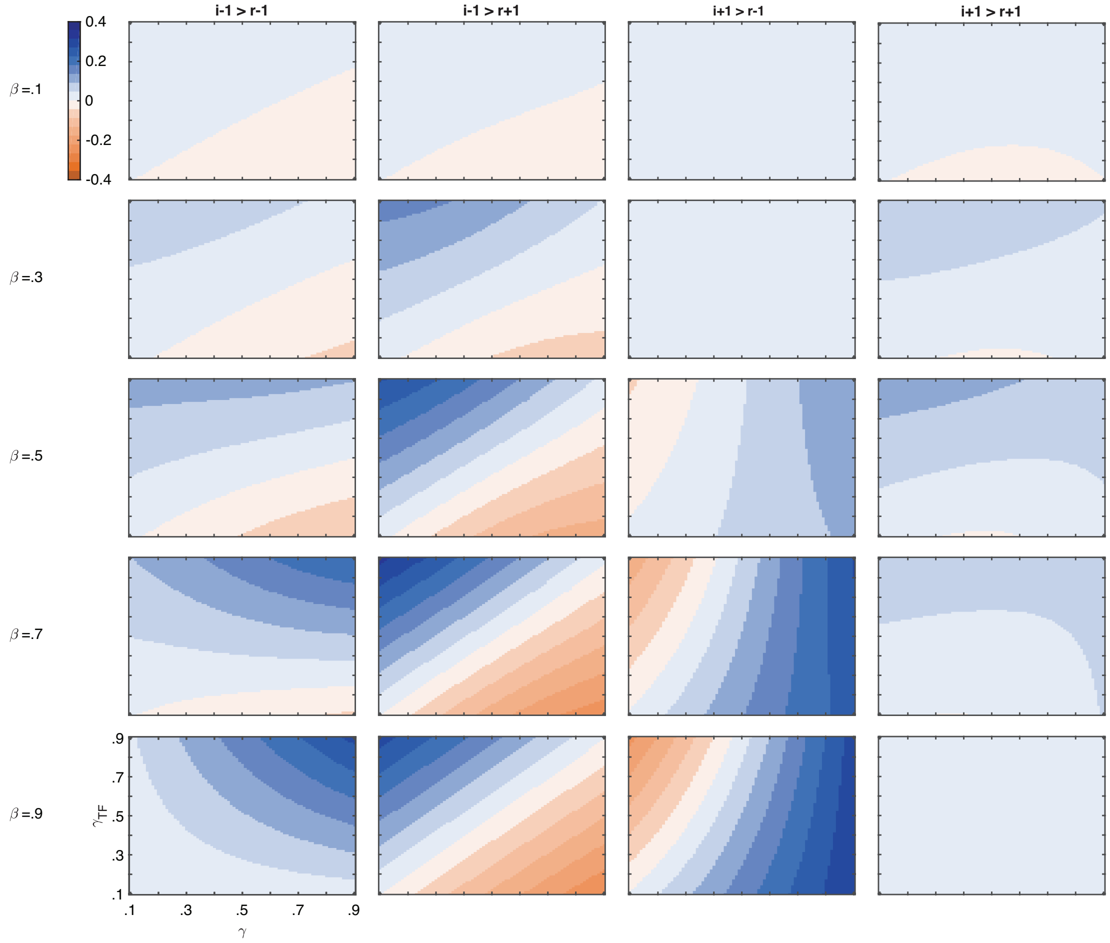

Note. The cue strength of the repeated item from each presentation neighbor was calculated based on presenting the repeated item six items apart, then presenting a presentation neighbor ten items later. Each plot shows the difference in cue strength of a pair of first- versus second-presentation neighbors across a range of values for $\beta, \gamma, \gamma_{TF}$. The color bar indicates the numerical difference of the cue strength for the first minus second-presentation neighbor: Blue indicates a greater cue strength from the first than second-presentation neighbor, and orange indicates a greater cue strength from the second- than first-presentation neighbor.

Figure S5

Difference in Context Cue Strength of Presentation Neighbors

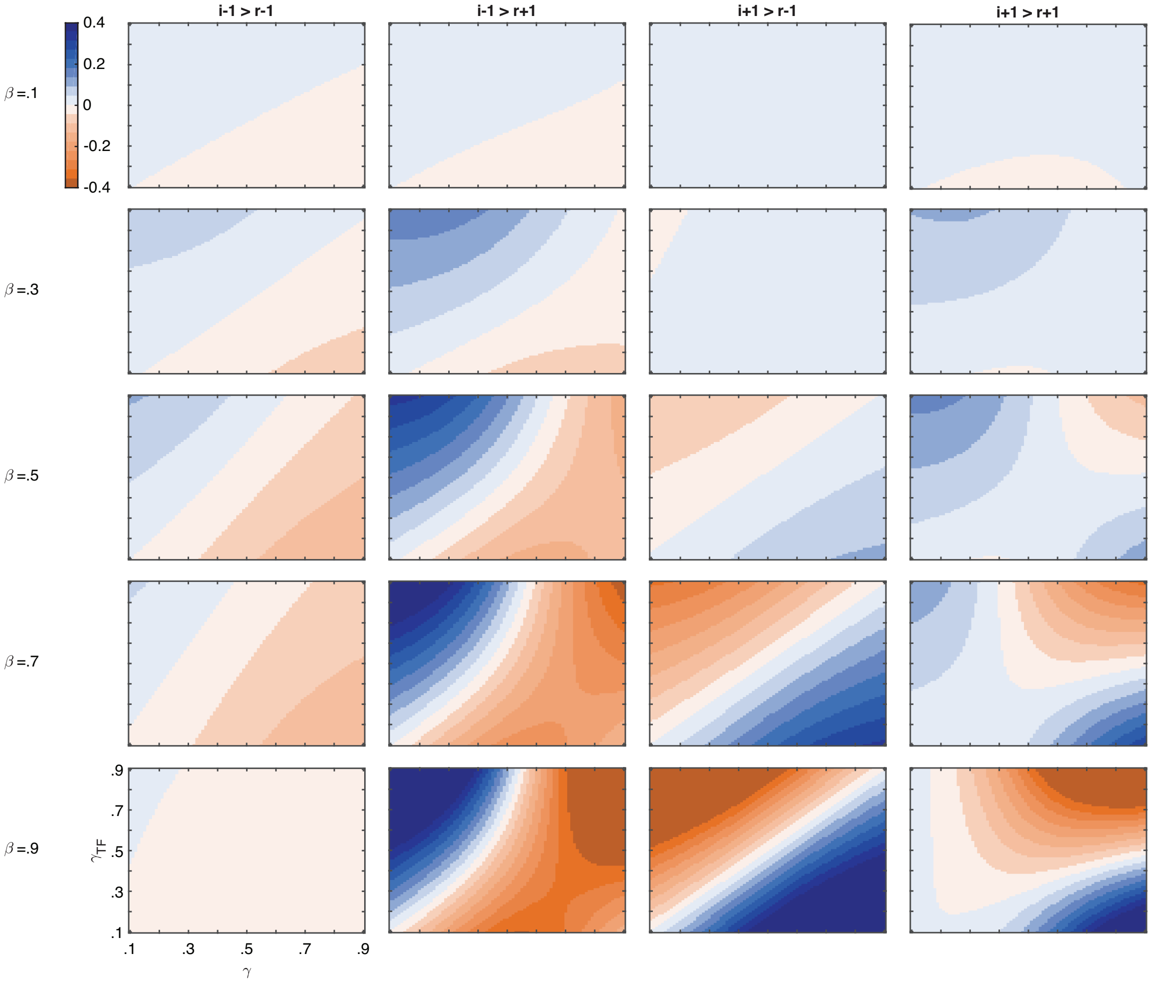

Note. The cue strength of the repeated item from each presentation neighbor was calculated based on presenting the repeated item six items apart, then presenting a presentation neighbor ten items later. Each plot shows the difference in cue strength of a pair of first versus second-presentation neighbors across a range of values for $\beta, \gamma, \gamma_{TF}$. The color bar indicates the numerical difference of the cue strength for the first- minus second-presentation neighbor: Blue indicates a greater cue strength from the first- than second-presentation neighbor, and orange indicates a greater cue strength from the second than first-presentation neighbor.

Figure S6

Difference in Context Cue Strength by Lag

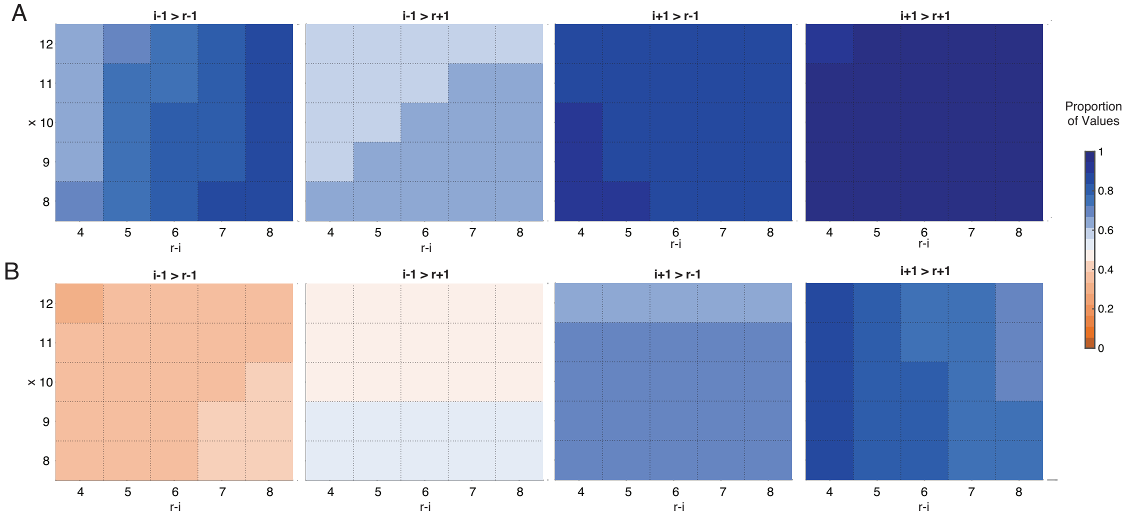

Note. Each panel plots the proportion of parameter sets varied across $\beta, \gamma, \gamma_{TF} \in [0.1, 0.9]$ with cue strengths satisfying the inequality at the top of the graph, where blue colors indicate a majority of values are greater for the first-presentation neighbors. (A) Simulations with $\Delta \mathbf{M}^{TF}_{exp} = (\Delta \mathbf{M}^{FT}_{exp})^\top = \mathbf{f}_i \mathbf{t}_i^\top$. (B) Simulations with $\Delta \mathbf{M}^{TF}_{exp} = (\Delta \mathbf{M}^{FT}_{exp})^\top = \mathbf{f}_i \mathbf{t}_{i-1}^\top$.

sumptions, lower values of the context drift rate β yielded lower differences in cue strengths for the first- than second-presentation neighbors. Lower values of β minimize the context drift rate, increasing the similarity among items. This approximates the boundary condition of retrieved context model to incorporate the assumption of context variability. Lower values of β also serve to minimize the context retrieval rate of the context associated with the first presentation of the repeated item ($\mathbf{t}_i$) during its second presentation ($\mathbf{t}_r$). Thus, this minimizes the core model assumption of context-based study-phase retrieval. Taken together, this finding highlights the core assumptions of retrieved context models to predict the increased associations and cue strengths of first-presentation over second-presentation neighbors.

These assumptions can also break down the other extreme—when both β and γ are very high—which can serve to overwrite a repeated item's context at second presentation with its context from the first. As a result, much of the advantage of the repeated item was then attributed to the second occurrence of the repeated item, providing greater cue strength to $r + 1$. These differences are more pronounced when each item is associated to the previous state of context, because the item $r + 1$ is associated to the repeated item's context, $\mathbf{t}_r$. Nonetheless, models typically do not best fit the data with all parameters close to the boundary values used here (e.g., Lohnas et al., 2015; Pazdera & Kahana, 2023; Polyn et al., 2009; Sederberg et al., 2008). Similarly, in the case of $i + 1$ versus $r - 1$, a low value of context retrieval rate (γ) exacerbates higher β values, causing context to drift further away from $i + 1$ yet share more context with its preceding neighbor $r - 1$.

Other commonalities among simulations include that results were relatively consis-

Figure S7

Recall Probability as a Function of Serial Position and Repetition Condition

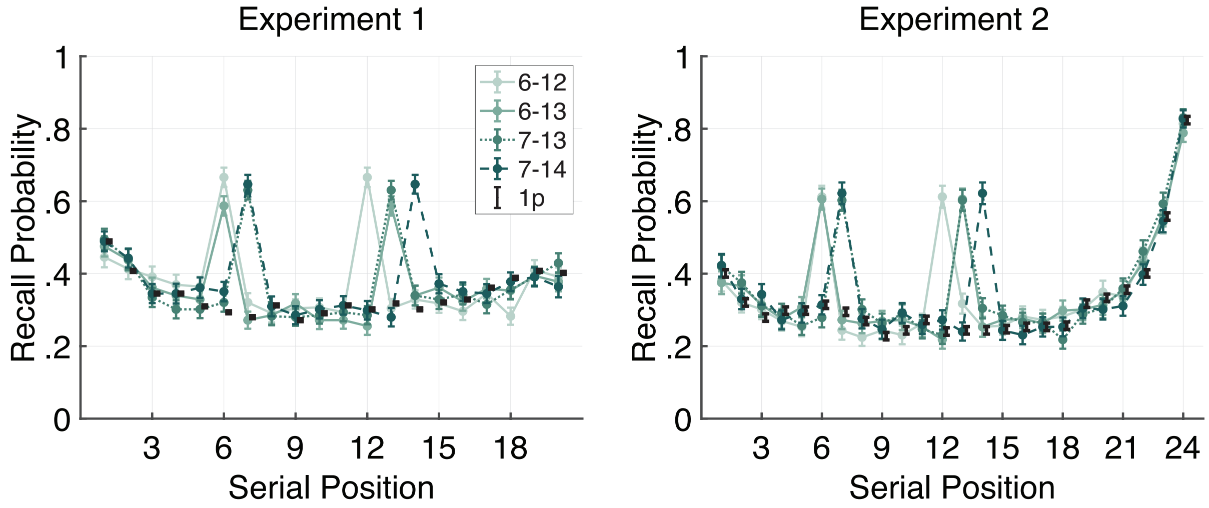

Note. In the legend, "A-B" indicates the two serial positions A and B at which the single list item was repeated. 1p = Lists with once-presented items only. Errorbars are standard error of the mean.

tent across lags. In addition, the cue strengths favoring first-presentation neighbors were generally weaker, in value and in proportion, when each item was associated to the previous rather than the current state of context. In actuality, context may be associated more continuously with the current or previous state of context, rather than the two extreme cases considered here, and this remains a future direction to explore.

Assuming that memory representations are consistent with CMR, another future direction is to acquire more transitions per participant, as this could help to dissociate the four pairwise comparisons of neighbors and thus which set of model mechanisms and parameters best account for the present data. The set of parameters producing the greatest cue strength advantage for first-presentation neighbors did not exhibit consistent patterns for all neighbors, yet this underscores the importance of considering the overall set of pairwise comparisons rather than a specific pair. That is, even if a parameter set did not exhibit greater first-presentation neighbor cue strength for one pair, other pairwise comparisons often still favored the first-presentation neighbor. Further, characterizing the favored cue strength for a single presentation pair could elucidate which model parameters and mechanisms best account for the results. However, critically the current simulations suggest that even with some variability in how CMR is actualized and across cue pairs, retrieved context models generally predict greater cue strengths for first-presentation than second-presentation neighbors.

Behavioral control analyses

Recall probability by serial position and repetition type

Overall, the serial position curves are relatively consistent between conditions, and recall of midlist positions is approximately equivalent. Comparing recall probability at the pairs of serial positions preceding the repeated item (3 vs. 4, 4 vs. 5 for lists with the first repetition at serial position 6; 4 vs. 5, 5 vs. 6 for lists with the first repetition at serial position 7) t-tests yielded no differences in recall probability, uncorrected $p's > 0.2$. This suggests that in the lists with repeated items, participants boasted similar levels of encoding ability by the time they reached the repeated items. Taken together these results suggest that fluctuations in attention or fatigue should be similar across the two serial positions at which the repetitions occurred. More generally, recall advantages at specific serial positions are less likely to drive the differences in recall transitions between first- and second-presentation neighbors (see also *Transition analyses on data sets with lists of once-presented items*).

Recall transition matrices as a function of serial position and lag

To better characterize the contributions of transitions to repeated items, Tables S7,S8 provide transitions as a function of serial position. For the experimental lists, transitions are counted based on the serial positions of the repeated item, with the first serial position as either 6 or 7 and the second presentation at serial position 12, 13 or 14. For the control lists, all recalled items are included for each set of serial positions, and thus includes transitions to items at serial position 6 and 7 for the third row, and serial position 12, 13 and 14 for the second row. (Note that these tables exclude when participants transition to the repeated item from an error, or when the repeated item is the first recalled item.)

Although a lower number of observations per cell prevents performing statistics on these numbers, nonetheless it is worth noting that among the presentation neighbors of the repeated item, there are more transitions from first-presentation neighbors. Further, with one exception there were more transitions to the repeated item, or to a matched serial position at lag=+1 than lag=-1, consistent with the forward asymmetry effect seen in standard free recall and with CMR predictions (Healey et al., 2019; Polyn et al., 2009).

Table S7

Experiment 1 Transitions to Serial Positions of the Repeated Item

List Type	Serial Positions	Lag							
		≤ -4	-3	-2	-1	1	2	3	≥ 4
Experimental	6,7	323	31	29	41	107	56	42	105
Experimental	12,13,14	145	25	34	35	47	24	26	398
Control	6,7	63	6	13	15	34	18	10	28
Control	12,13,14	57	14	15	22	65	24	17	85

Table S8

Experiment 2 Transitions to Serial Positions of the Repeated Item

List Type	Serial Positions	Lag							
		≤ -4	-3	-2	-1	1	2	3	≥ 4
Experimental	6,7	381	21	27	38	72	26	33	70
Experimental	12,13,14	221	24	31	39	33	25	15	280
Control	6,7	67	7	7	19	33	14	10	13
Control	12,13,14	70	3	7	19	33	14	9	46

Serial positions of transitions from the repeated item may also be of interest and are provided in Tables S9,S10 (excluding when participants transition from the repeated item to an error, or when the repeated item is the last recalled item). However, as described in the main Introduction the results from the present studies pose difficulties in distinguishing between theories. If one of the presentation neighbors was recalled when transitioning to the repeated item (e.g., 5), the transition from a repetition to a remaining neighbor (e.g., 13) may only occur in the absence of neighbors with stronger associations. In the case of transitions between the repeated item which both favor first- or second-presentation neighbors (e.g., 11, 6/12, 13), this may reflect lingering cue information from the item transitioned to the repeated item (11) also benefiting the item transitioned from the repeated item (e.g., Lohnas & Kahana, 2014). Distinguishing between these possibilities, with higher powered studies and accounting for recall availability, is a notable direction for future work.

Table S9

Experiment 1 Transitions from Serial Positions of the Repeated Item

List Type	Serial Positions	Lag							
		≤ -4	-3	-2	-1	1	2	3	≥ 4
Experimental	6,7	77	39	30	46	68	48	31	391
Experimental	12,13,14	322	35	28	40	82	46	39	138
Control	6,7	15	9	7	9	32	8	5	72
Control	12,13,14	72	11	13	25	61	19	14	45

Table S10

Experiment 2 Transitions from Serial Positions of the Repeated Item

List Type	Serial Positions	Lag							
		≤ -4	-3	-2	-1	1	2	3	≥ 4
Experimental	6,7	66	20	21	37	69	23	25	313
Experimental	12,13,14	243	33	17	28	47	20	25	161
Control	6,7	13	2	11	19	33	9	8	52
Control	12,13,14	37	11	5	22	34	16	5	40

Transition analyses on data sets with lists of once-presented items

To control for contributions of serial position, output position and lag recency to transitions to repeated items, I conducted transition analyses to matched serial positions in lists of once-presented items. The current studies did not collect enough data per participant for such analyses, as there were only two lists per participant. Instead, I conducted analyses on data sets with similar methods yet more lists per participant.

Methods

More complete methods are provided in the original publications, but critical details are provided below.

Experiment 1 Control: Bridge, 2006.

Participants and Sampling Procedures: One hundred and nineteen participants were Syracuse University undergraduates who participated for course credit. Because some participants never recalled items at serial positions of interest (see Analyses), 102 participants were included in the final analysis.

Materials: Participants studied lists of comprised nouns drawn without replacement from the Toronto Word Pool (Friendly et al., 1982).

Procedure: Participants performed free recall of lists with length 25. Most participants had 18 lists ($N = 77$), however the remaining participants completed fewer lists (14–17, $M = 16.8$). During each word presentation, participants were given 1,100 ms to judge whether the word was “concrete” or “abstract”. A judgment response initiated a 200 ms ISI period. After the presentation of the last item, for 30 s participants performed arithmetic problems of the form $A + B + C = ?$, where A, B, C were positive, single-digit integers. After the distractor task, participants were given 60 s to vocally recall the just-presented list.

Experiment 2 Control: Lohnas & Kahana 2014.

Participants and Sampling Procedures: This data set is composed of three sets of participants while data collection was ongoing, some reported for the first time in Lohnas and Kahana, 2014 all with data collected at the University of Pennsylvania (Lohnas & Kahana, 2014; Lohnas et al., 2011; Polyn et al., 2009). The original data set has 106 participants who performed 1–6 sessions each, yet because some participants never recalled the items at serial positions of interest (see Analyses) for the included lists (see Procedure), 102 participants were included in the final analysis.

Materials: Lists of words were drawn for each participant from the word association spaces norms (WAS; Steyvers et al., 2004) without replacement for each session.

Procedure: Participants performed immediate free recall of lists with length 24 in which each word was presented visually for 3,000 ms, with an 800 ms ISI. The analyses here include only single-task lists (6–36 lists per participant, $M = 20.5$). For these lists, participants performed one of two encoding tasks for all list items, either a size or animacy judgment of the current word. After the final item, participants were given 90 s to vocally recall the just-presented list.

Analyses. For each participant, the lists were assigned pseudorandomly to one of the four repetition serial position conditions (6,12; 6,13; 7,13; 7,14) such that the lists were distributed across the four options as uniformly as possible (e.g., rather than randomly

Figure S8

Results of Control Conditions

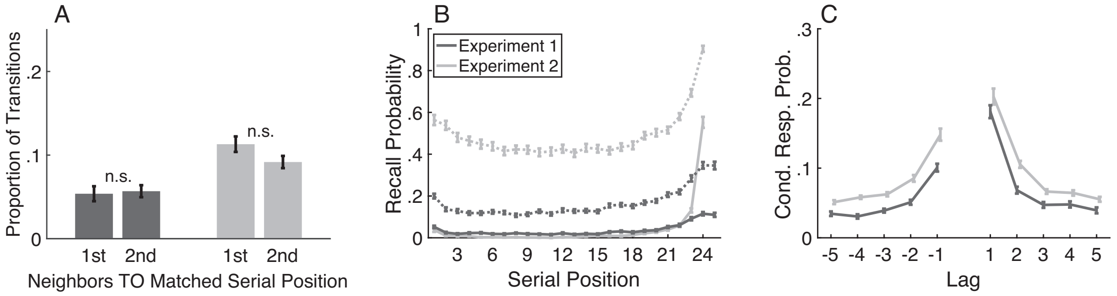

Note. A. Proportion of recall transitions to neighbors of serial positions matched to repeated items but in control studies using lists of once-presented items. B. Recall probability of the first recall (solid lines) and across all output positions (dashed lines). C. Conditional response probability as a function of lag. For Experiment 2, this excludes the first three output positions (cf. Kahana, 1996). n.s. = $p > .05$. Errorbars are standard error of the mean.

drawing with replacement from the four conditions, if the number of lists for a participant was divisible by four, then the participant was assigned an equal number of lists from each condition). For each list, the first recall in the sequence corresponding to either of the serial positions of the repeated item (e.g., 6, 12) was found. If the recalled item was the first instance of the matched repetition (here, 6) and the serial position of the item preceding that recall had absolute lag 1 from the first serial position (5, 7), then a transition from the first "presentation" was tallied. Similarly, if the recalled item was the second instance of the matched repetition (12) then a transition from the second "presentation" was tallied for a neighbor to the second serial position (11, 13). Then, the proportion of transitions was determined across all lists with a recall from either of the serial positions matching a repeated item. The proportions were calculated for 10 sets of pseudorandom assignments of serial position pairs, then all 10 were averaged within participant to give an estimate of the baseline proportion for each participant.

Results

In both data sets, transitions were not significantly different between matched first- and second-presentation neighbors (Figure S8A), Experiment 1 control: $M = 0.0538\ vs.\ 0.0568, SEM = 0.0117, t(101) = 0.259, p = 0.80, CI = [-0.0263, 0.0202], d = 0.037$; Experiment 2 control: $M = 0.113\ vs.\ 0.0917, SEM = 0.0110, t(101) = 1.93, p = 0.056, CI = [-0.000545, 0.0432], d = 0.25$.[2]

The control studies and the present studies share similar profiles of serial position,

[2] Although the effect size is weak for the Experiment 1 control, this does suggest that there are not reliable effects of transitions in these data. Further suggestive of this point, the effect in this control experiment was significantly less than the effect than Experiment 1: $M = 0.0648\ vs. - 0.00303, SEM = 0.0143, t(284) = 2.27, p = 0.024, CI = [0.00903, 0.127], d = 0.24$. Providing further assurance of this point and of any concerns about more recency in recall initiation, an exploratory post-hoc analysis on the subset of participants who were more likely to initiate recall with the first than last list item also yielded a larger effect size: $M = 0.0746\ vs.\ 0.0577, SEM = 0.0263, t(26) = 0.640, p = 0.53, CI = [-0.0373, 0.0710], d = 0.19$.

probability of first recall and contiguity effects (Figure S8B,C). Further, there were no significant differences in recall probability at serial positions preceding the serial positions of the first repetition serial position, suggesting that these serial positions were not a part of the early-list recall advantage or primacy effect (i.e., comparing 3 vs. 4, 4 vs. 5, 5 vs. 6, uncorrected $p's > 0.2$). Thus, recall among once-presented items by serial position, output position and temporal contiguity most likely do not drive the effects of transitions to a repeated item. Instead, the main difference between the study pairs is the absence or presence of a repeated item, and thus most likely the elevated transitions to the repeated item reflect its unique properties.

References

Bridge, D. (2006). Memory and cognition: What difference does gender make? *Unpublished Honor's thesis, Syracuse University.*

Friendly, M., Franklin, P. E., Hoffman, D., & Rubin, D. C. (1982). The Toronto Word Pool: Norms for imagery, concreteness, orthographic variables, and grammatical usage for 1,080 words. *Behavior Research Methods and Instrumentation*, *14*, 375–399. https://doi.org/10.3758/BF03203275

Gershman, S. J., Moore, C. D., Todd, M. T., Norman, K. A., & Sederberg, P. B. (2012). The successor representation and temporal context. *Neural Computation*, *24*(6), 1553–1568. https://doi.org/10.1162/NECO_a_00282

Healey, M. K., Long, N. M., & Kahana, M. J. (2019). Contiguity in episodic memory. *Psychonomic Bulletin & Review*, *26*(3), 699–720. https://doi.org/10.3758/s13423-018-1537-3

Kahana, M. J. (1996). Associative retrieval processes in free recall. *Memory & Cognition*, *24*(1), 103–109. https://doi.org/10.3758/BF03197276

Lohnas, L. J., & Kahana, M. J. (2014). Compound cuing in free recall. *Journal of Experimental Psychology: Learning, Memory, and Cognition*, *40*(1), 12–24. https://doi.org/10.1037/a0033698

Lohnas, L. J., Polyn, S. M., & Kahana, M. J. (2011). Contextual variability in free recall. *Journal of Memory and Language*, *64*(3), 249–255. https://doi.org/10.1016/j.jml.2010.11.003

Lohnas, L. J., Polyn, S. M., & Kahana, M. J. (2015). Expanding the scope of memory search: Intralist and interlist effects in free recall. *Psychological Review*, *122*(2), 337–363. https://doi.org/10.1037/a0039036

Lohnas Siegel, L., & Kahana, M. J. (2014). A retrieved context account of spacing and repetition effects in free recall. *Journal of Experimental Psychology: Learning, Memory, and Cognition*, *40*, 755–764. https://doi.org/10.1037/a0035585

Pazdera, J. K., & Kahana, M. J. (2023). Modality effects in free recall: A retrieved-context account. *Journal of Experimental Psychology: Learning, Memory, and Cognition*, *49*(6), 866–888. https://doi.org/10.1037/xlm0001140

Polyn, S. M., Norman, K. A., & Kahana, M. J. (2009). A context maintenance and retrieval model of organizational processes in free recall. *Psychological Review*, *116*, 129–56. https://doi.org/10.1037/a0014420

Sederberg, P. B., Howard, M. W., & Kahana, M. J. (2008). A context-based theory of recency and contiguity in free recall. *Psychological Review*, *115*(4), 893–12. https://doi.org/10.1037/a0013396

Steyvers, M., Shiffrin, R. M., & Nelson, D. L. (2004). Word association spaces for predicting semantic similarity effects in episodic memory. In A. F. Healy (Ed.), *Cognitive psychology and its applications: Festschrift in honor of Lyle Bourne, Walter Kintsch, and Thomas Landauer.* American Psychological Association. https://doi.org/10.1037/10895-018
